# AmrZ Regulates Swarming Motility Through Cyclic di-GMP-Dependent Motility Inhibition and Controlling Pel Polysaccharide Production in *Pseudomonas aeruginosa* PA14

**DOI:** 10.3389/fmicb.2019.01847

**Published:** 2019-08-14

**Authors:** Lingli Hou, Alexander Debru, Qianqian Chen, Qiyu Bao, Kewei Li

**Affiliations:** ^1^Department of Microbiology and Immunology, Key Laboratory of Laboratory Medicine, Ministry of Education, School of Laboratory Medicine and Life Sciences, Wenzhou Medical University, Wenzhou, China; ^2^Scientific Research Center of Wenzhou Medical University, Wenzhou, China

**Keywords:** *Pseudomonas aeruginosa*, AmrZ, swarming motility, cyclic di-GMP, GcbA, FlgZ, PA14_56180, exopolysaccharide

## Abstract

Swarming is a surface-associated motile behavior that plays an important role in the rapid spread, colonization, and subsequent establishment of bacterial communities. In *Pseudomonas aeruginosa*, swarming is dependent upon a functional flagella and aided by the production of biosurfactants. AmrZ, a conserved transcription factor across pseudomonads, has been shown to be a global regulator of multiple genes important for virulence and ecological fitness. In this study, we expand this concept of global control to swarming motility by showing that deletion of *amrZ* results in a severe defect in swarming, while multicopy expression of this gene stimulates swarming of *P. aeruginosa*. Mechanistic studies showed that the swarming defect of an *amrZ* mutant does not involve changes of biosurfactant production but is associated with flagellar malfunction. The ∆*amrZ* mutant exhibits increased levels of the second messenger cyclic di-GMP (c-di-GMP) compared to the wild-type strain, under swarming conditions. We found that the diguanylate cyclase GcbA was the main contributor to the increased accumulation of c-di-GMP observed in the ∆*amrZ* mutant and was a strong inhibitor of flagellar-dependent motility. Our results revealed that the GcbA-dependent inhibition of motility required the presence of two c-di-GMP receptors containing a PilZ domain: FlgZ and PA14_56180. Furthermore, the ∆*amrZ* mutant exhibits enhanced production of Pel polysaccharide. Epistasis analysis revealed that GcbA and the Pel polysaccharide act independently to limit swarming in Δ*amrZ*. Our results support a role for AmrZ in controlling swarming motility, yet another social behavior besides biofilm formation that is crucial for the ability of *P. aeruginosa* to colonize a variety of surfaces. The central role of AmrZ in controlling these behaviors makes it a good target for the development of treatments directed to combat *P. aeruginosa* infections.

## Introduction

Swarming is a surface-associated mode of motility that involves rapid and coordinated movement of a bacterial population across viscous semisolid surfaces (Kearns, 2010; Partridge and Harshey, 2013). In the opportunistic human pathogen *Pseudomonas aeruginosa* (Silby et al., 2011), this type of movement requires the presence of a functional flagella to mediate actual movement and is aided by the production of biosurfactants such as rhamnolipids to overcome the surface tension between cells and the surrounding environment (Kohler et al., 2000; Kearns, 2010). Since human mucosal surfaces, such as the epithelial surfaces of the lung represents a viscous environment analogous to the conditions that promote swarming *in vitro*, swarming motility is considered clinically relevant (Yeung et al., 2012). Moreover, there is evidence that swarming does not simply enable the bacterium to move but is also a complex lifestyle adaptation in response to various environmental cues, resulting in substantial changes in metabolism (Yeung et al., 2009; Ghorbal et al., 2019), increased virulence gene expression and antibiotic resistance (Overhage et al., 2008; Tremblay and Deziel, 2010). Thus, investigation of the key molecules and mechanisms regulating swarming motility is important for the development of treatment against this bacterium.

Cyclic di-GMP (c-di-GMP) is a nearly ubiquitous bacterial second messenger that regulates diverse cellular processes and is of key importance for modulating transitions between motile and sessile lifestyles important for acute and chronic infections, respectively (Hengge, 2009; Romling et al., 2013). Like other bacteria, *P. aeruginosa* also uses c-di-GMP to create an inverse regulation of biofilm formation and swarming motility (Valentini and Filloux, 2016; Baker et al., 2019). According to a well-established model, increased levels of c-di-GMP have a negative effect on swarming and are correlated with a sessile lifestyle, while at low levels the bacteria can move and swarm away in search of better conditions (Ha and O’Toole, 2015; Yan et al., 2017). c-di-GMP is synthesized by diguanylate cyclases (DGCs) and is degraded by phosphodiesterases (PDEs). The *P. aeruginosa* genome encodes over 40 such enzymes that contribute to the steady-state levels of intracellular c-di-GMP (Ha and O’Toole, 2015). To exert its control, c-di-GMP binds to different classes of effector proteins or RNAs (Hengge, 2009). Currently, proteins with a PilZ domain represent the largest family of c-di-GMP effectors (Orr and Lee, 2016), and the highly conserved RXXXR and (D/N)XSXXG motifs in the PilZ domain are essential for c-di-GMP binding (Amikam and Galperin, 2006; Cheang et al., 2019). In the c-di-GMP-bound state, PilZ domain proteins regulate diverse cellular processes such as virulence, biofilm formation and flagellum-dependent motility (Baker et al., 2016; Xu et al., 2016a,b).

AmrZ, a transcription factor that belongs to the ribbon-helix-helix (RHH) family of DNA-binding proteins (Pryor et al., 2012), was originally described as AlgZ for its ability to activate alginate production in *P. aeruginosa* (Baynham and Wozniak, 1996). It was later changed to AmrZ (alginate and motility regulator) because of its positive role in the regulation of Type IV Pili biogenesis and twitching motility (*a pili dependent motility*) (Baynham et al., 2006). Its implication in regulation of motility was then expanded by the finding that AmrZ acts as a negative regulator of flagellum biosynthesis in mucoid, nonmotile *P. aeruginosa* isolates from CF patients (Tart et al., 2006). In this case, AmrZ is expressed at high levels and directly represses transcription of the flagellar master regulator FleQ and thus flagellum biosynthesis (Tart et al., 2006). However, in nonmucoid strains, the synthesis of flagellin is similar between wild-type and the *amrZ* deletion mutant (Baynham et al., 2006), and the role of AmrZ in flagellum-driven motility is still unclear. Additionally, AmrZ inhibits the production of the Psl polysaccharide by directly repressing transcription of the *psl* operon, which is involved in *P. aeruginosa* biofilm development (Jones et al., 2013, 2014). Recent studies have shown that AmrZ functions as a global regulator (Jones et al., 2014; Martinez-Granero et al., 2014; Allsopp et al., 2017) and one of the AmrZ regulated targets is c-di-GMP signaling. It was shown that the Δ*amrZ* mutant displays elevated levels of c-di-GMP and forms hyper biofilms compared with the wild-type strain PAO1 (Jones et al., 2014). Considering that c-di-GMP levels are higher in Δ*amrZ* and that elevated levels of c-di-GMP inhibit motility (McCarter and Gomelsky, 2015), we hypothesized motility might be impaired in Δ*amrZ* cells.

Here, we investigated the role of AmrZ in swarming and the underlying molecular mechanism of this association. We find that AmrZ positively controls swarming motility of *P. aeruginosa* and the swarming deficiency of a ∆*amrZ* mutant is associated with flagellar malfunction but not insufficient biosurfactant production. We demonstrate that the AmrZ-mediated regulation of swarming involves a c-di-GMP signaling module consisting of the DGC GcbA and the c-di-GMP receptors FlgZ and PA14_56180. We also uncovered negative regulation of Pel polysaccharide production by AmrZ. This regulation does not involve changes in *pel* expression and modulates the ability of *P. aeruginosa* to swarm.

## Materials and Methods

### Bacterial Strains, Plasmids, and Culture Conditions

The strains and plasmids used in this study are listed in Supplementary Table S1. *P. aeruginosa* strain UCBPP-PA14 (abbreviated *P. aeruginosa* PA14) was used as the parental strain, unless otherwise stated. *Escherichia coli* DH5α was used as the host for DNA cloning. *P. aeruginosa* and *E. coli* strains were routinely cultured in Lysogeny Broth (LB) medium (10 g of tryptone, 5 g of yeast extract, and 5 g of NaCl per liter, pH 7.0) or on LB Agar (LB medium containing 1.5% [w/v] agar) at 37°C unless otherwise noted. For expression plasmids with the P*_BAD_* promoter, arabinose was added to cultures at a 0.2% final concentration. Where necessary, 50 μg/ml gentamicin, 150 μg/ml carbenicillin, and 50 μg/ml tetracycline were used for *P. aeruginosa* and 100 μg/ml ampicillin, 50 μg/ml kanamycin, 10 μg/ml gentamicin, and 10 μg/ml tetracycline were used for *E. coli*.

### Construction of Strains and Plasmids

In-frame deletion mutants were constructed by allelic exchange using the sucrose counter-selection system as previously described with the *P. aeruginosa* suicide vector pEX18Tc (Hoang et al., 1998). Mutant strains were confirmed by PCR analysis of genomic DNA. In addition, single-copy chromosomal complementation of the *amrZ* mutation was accomplished by introducing *amrZ* under the control of its native promoter into pUC18T-mini-Tn*7*T-Gm (Choi and Schweizer, 2006). Overexpression was accomplished by placing the respective genes under the control of the constitutive *lac* promoter in pUCP20 (West et al., 1994). For complementation of the *flgZ or PA14_56180* mutant in the GcbA-overexpressing background with the respective C-terminally FLAG-tagged WT and mutated proteins, primers were designed to contain the open reading frame and ribosome-binding site with the indicated oligonucleotides (Supplementary Table S2) and expression of the respective genes was under the control of the arabinose-inducible P*_BAD_* promoter in the pUC18T-mini-Tn*7*T-Gm-BAD integration vector. pUC18T-mini-Tn*7*T-Gm-BAD was created by inserting a NsiI-SacI digested *araC*-P*_BAD_* cassette from pBAD18 (Guzman et al., 1995) into pUC18T-mini-Tn*7*T-Gm cleaved at its NsiI and SacI sites in the MCS. The identity of vector inserts was verified by PCR and sequencing. Plasmids were introduced into *P. aeruginosa* by electroporation (Choi et al., 2006). The primers used for strain construction are listed in Supplementary Table S2.

### Motility Assays

Swarming motility assays were performed as previously described on 0.5% (w/v) agar M8 plates (Ha et al., 2014) supplemented with 0.2% (w/v) glucose, 0.5% (w/v) casamino acids, and 1 mM MgSO_4_ (Kohler et al., 2000; Kuchma et al., 2007). After solidification, plates were briefly dried at room temperature and spot inoculated with 2.5 μl aliquots taken directly from overnight LB cultures. Swarming plates were incubated face up in stacks of no more than two at 37°C for 16–18 h. To quantify the degree of swarming, an image of the swarming plate was captured with a digital camera and percent coverage of the plate was measured by comparing swarming pixels with total plate pixels using Adobe Photoshop CS6. Swim plates were identical to swarm plates except that it was solidified with 0.3% (w/v) agar (Caiazza et al., 2005). Swimming assays were carried out as reported previously and incubated at 30°C for 18–20 h (Murray et al., 2010; Li et al., 2017). Experiments were repeated in triplicate and the data are presented as averages over three replicate plates.

### Quantitative Real-Time Reverse-Transcription PCR Analysis

Quantitative real-time reverse-transcription PCR (qRT-PCR) was used to determine the gene expression levels of wild-type and indicated mutant strains. Planktonic cells were grown in liquid swarming medium to the exponential phase (OD_600_ = 0.9–1.0) under shaking conditions (Overhage et al., 2008). Cells growing on swarming plate were harvested from the tip of migrating tendrils or from the center of swarming colonies and were collected directly into RNAlater reagent (Qiagen, Germany) (Overhage et al., 2008; Tremblay and Deziel, 2010). Biofilms were grown directly on 6-well polystyrene microplates (Costar #3506) in liquid swarming medium and incubated as a static culture for 6 and 24 h at 37°C (Friedman and Kolter, 2004; Petrova et al., 2014; Price et al., 2016). At each time point, unattached bacteria were removed by aspiration and the remaining biofilms were washed once with PBS and disrupted with a cell scraper. RNA was extracted using the RNAprep pure Kit (TIANGEN Biotech, Beijing, China) according to the manufacturer’s instructions. To remove residual DNA, RNA was further treated with DNase I, repurified with an RNAclean Kit (TIANGEN), and was confirmed to be free of DNA by PCR. cDNA was synthesized with a PrimeScript RT Reagent Kit (Perfect Real Time) (TaKaRa, Liaoning, China) using random hexamer primers. qRT-PCR was performed on the Bio-Rad CFX96 Touch real-time PCR detection system using SYBR® Premix Ex Taq™ (Tli RNaseH Plus) mix (Takara). The mRNA levels were normalized by the geometric mean of *PA14_26910* (*PA2875*) and *PA14_20860* (*PA3340*) transcript levels (Costaglioli et al., 2014), and relative gene expression was calculated using the 2^−ΔΔCt^ method (Livak and Schmittgen, 2001).

### Rhamnolipid Production and Drop Collapse Assays

Rhamnolipid biosynthesis was analyzed *via* the cetyltrimethylammonium bromide (CTAB)-methylene blue plate method as previously described (Siegmund and Wagner, 1991; Caiazza et al., 2005). Briefly, 2.5 μl of overnight LB cultures were spot inoculated onto plates consisting of the same base medium as for the swarming assays but supplemented with 0.02% (w/v) cetyltrimethylammonium bromide (CTAB), 0.0005% (w/v) methylene blue, and solidified with 1.5% (w/v) agar. Plates were incubated face up at 37°C for 24 h, and then incubated for an additional 24 h at room temperature. Rhamnolipid production was determined by measuring the diameter of the dark blue halo surrounding a colony. To detect the production of the rhamnolipid precursor HAA, drop collapse assays were performed on 0.22-μm membrane filtered supernatants prepared from LB overnight cultures at 30°C (Murray and Kazmierczak, 2008). Filtered supernatant was serially diluted (1:1) with sterile water in a 96-well plate, and 30 μl of each dilution was spotted onto the circles on the underside of the lid of a Corning 96-well plate, allowing surfactant activity to be measured by the spread of the droplet as described (Caiazza et al., 2005; Murray and Kazmierczak, 2008). Each assay was repeated at least three times.

### Transmission Electron Microscopy

Transmission electron microscopy (TEM) was used to visualize cell morphology and the presence of flagella as described previously (Kohler et al., 2000; Rashid and Kornberg, 2000). Briefly, bacteria at the advancing swarm edge were gently scraped and suspended in 2.5% (v/v) glutaraldehyde in 0.1 M PBS (pH 7.2) and the fixation process started at the same time. Fifteen minutes later, carbon-coated copper grids were placed on top of a drop of the cell suspension for 30 s to allow the adhesion of bacterial cells. Grids were then stained for 2 s with 2% uranyl acetate (w/v) and washed twice for 10 s in a drop of distilled water. The grid was air dried and examined on a Hitachi H-7500 transmission electron microscope at calibrated magnifications. Approximately 200 cells were counted to determine the percentage of flagellated cells for each strain.

### Reversal Rate Measurements

Reversal rate measures the frequency at which a motile cell changes its direction (Toutain et al., 2005; Caiazza et al., 2007; Petrova et al., 2014). Bacteria expressing green fluorescent protein (GFP) were cultured overnight, sub-cultured and grown to the exponential phase (OD_600_ of 1.0) before observation. The GFP-expressing bacteria were generated by introduction of a multicopy plasmid (pUCP-*zsGreen1*, GFP^+^) that constitutively expresses ZsGreen1 GFP under the control of the *lac* promoter (Li et al., 2017). Cultures were then diluted 1:100 into M63 medium (Kuchma et al., 2007) supplemented with 0.2% (w/v) glucose and containing 3% (w/v, low-viscosity/swimming conditions) or 15% (w/v) Ficoll (high-viscosity/swarming conditions) (Toutain et al., 2005; Caiazza et al., 2007). Cells were monitored *via* fluorescence microscope (Eclipse Ni-U, Nikon) equipped with a 100×/1.45 oil objective lens and a Nikon DS-Fi1C camera. Real-time videos were captured using the NIS-Elements F Ver4.00.00 and Camtasia Studio V7.5 software package. The videos were subsequently analyzed to monitor individual cells for the number of times they reversed motility direction while within the field of view. Approximately 50 cells were measured for each strain and reversal rates are expressed as no. of reversals per cell per minute.

### Cyclic di-GMP Quantification

Intracellular levels of c-di-GMP were determined by LC-MS/MS as previously described with minor modifications (Merritt et al., 2010; Spangler et al., 2010; Chen et al., 2014). Swarm cells were washed off the surface of swarming plates with PBS by gently pipetting while slightly inclining the petri dish. Six swarming plates were used for each strain and for the swarming deficient *amrZ* mutant, each plate was inoculated with three colonies and 10 plates were used. Cell numbers were then normalized to an OD_600_ of 2.0, and 5 ml of this suspension was pelleted by centrifugation at 4°C for 10 min at 5,000 rpm. Pellets were resuspended in a small amount of supernatant, transferred to a pre-weighed 1.5 ml microcentrifuge tube, and then pelleted again. After removing the supernatant, cell pellets were vigorously resuspended in 250 μl of extraction buffer (acetonitrile/methanol/water [40/40/20, v/v/v] plus 0.1 M formic acid) and incubated at −20°C for 30 min. The cell debris was then centrifuged at 13,000 rpm for 5 min at 4°C, and 200 μl of supernatants containing the nucleotide extract were recovered into new tubes and neutralized with 4 μl of 15% NH_4_HCO_3_ (w/v) per 100 μl of sample. The resultant extracts and the tubes with cell debris were dried using a vacuum concentrator. The pellet weights were measured to get sample dry weight, and the dried extracts were resuspended in 200 μl of HPLC grade water and analyzed by LC-MS/MS to quantify the amount of c-di-GMP. Quantifications were performed in triplicate and the c-di-GMP levels were normalized to the dry weight of the cell pellet from which c-di-GMP was extracted and presented as pmol of c-di-GMP/mg of dry weight (Kuchma et al., 2012; Chen et al., 2014).

### Western Blot Analysis

Strains were cultured on swarming plates supplemented with 0.2% arabinose and appropriate antibiotics (Kuchma et al., 2015). Cells were harvested by gentle scraping with plastic coverslips and resuspended in PBS. Samples were normalized to equivalent number of bacteria cells by OD_600_ (Li et al., 2013). For Western blotting, whole-cell lysates were resolved by SDS-PAGE using 15% polyacrylamide gels. Proteins transferred to a polyvinylidene difluoride (PVDF) membrane were probed with an anti-FLAG antibody (Sigma, Shanghai, China) to detect FLAG-tagged FlgZ and PA14_56180 and mutant variants of these proteins. Signals were developed with a Western Lightning Plus-ECL kit (PerkinElmer, MA, USA).

### Congo Red-Binding Assays

To examine exopolysaccharide production, Congo red (CR)-binding assays were performed on tryptone (10 g/L) agar (1%) plates supplemented with CR (40 μg/ml) and Coomassie brilliant blue R (20 μg/ml) (Kong et al., 2013). Bacteria from LB-grown overnight cultures were diluted to an OD_600_ of 0.025 in PBS and a 2 μl volume was used for inoculation. Plates were incubated at 37°C for 24 h, followed by 4 days at room temperature before inspection (Kuchma et al., 2007; Colvin et al., 2012). To quantify the CR-binding levels (Andrews and Maurelli, 1992; Cangelosi et al., 1999), colonies were scraped from CR plates and suspended with vigorous vortexing in 600 μl of deionized water. After recording the OD_660_ of the suspension, cells were pelleted, resuspended in 200 μl of acetone, vortexed and the CR dye was extracted at room temperature for 2 h. Cells were then centrifuged, and the OD_488_ of the supernatant containing the extracted CR was measured. The relative Congo red-binding (RCRB) value was calculated by dividing the OD_488_ of the acetone extracts by the OD_660_ of the original cell suspension.

### Data Presentation and Statistical Analysis

The data are presented as the mean values ± standard deviations (mean ± SD). All significant differences were evaluated on SPSS program by using the Student’s *t*-test or one-way analysis of variance (ANOVA) followed by the Tukey’s test for multiple comparisons when applicable: ^*^p < 0.05; ^**^p < 0.01.

## Results

### AmrZ Is an Important Contributor to Swarming Motility in *P. aeruginosa*

**Figure 1:**
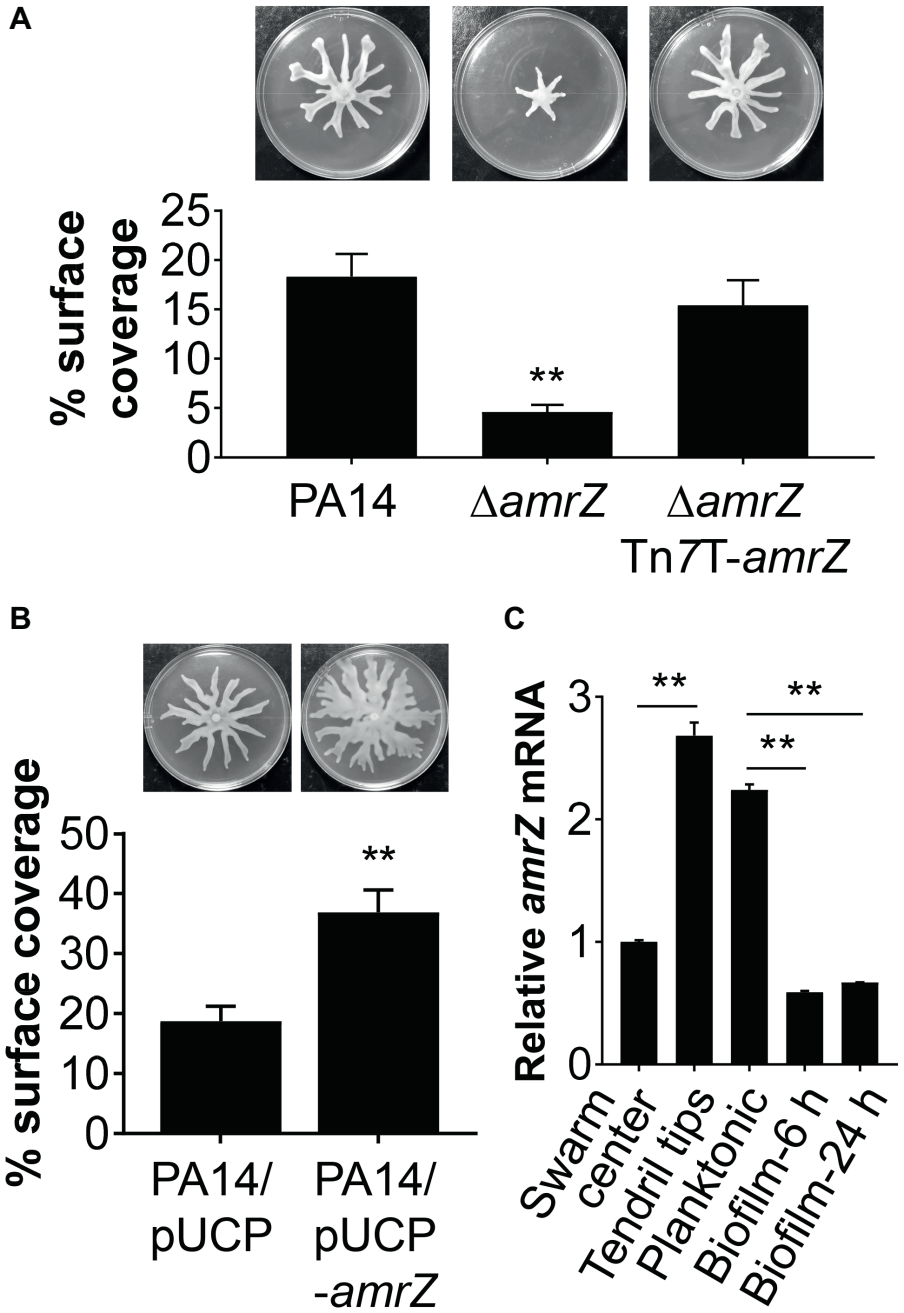
AmrZ plays a role in the regulation of swarming motility in *P. aeruginosa* PA14. **(A)** Swarming motility and average percentage of the surface coverage of WT PA14, the Δ*amrZ* mutant, and the single-copy complemented strain of the Δ*amrZ* mutant carrying an *amrZ* gene inserted into chromosome by mini-Tn*7*T. Error bars represent the standard deviations. **p < 0.01 compared with WT or complemented strains by Student’s *t*-test. **(B)** Swarm analysis and quantification of percent coverage of swarm plates for the WT strain carrying vector control (PA14/pUCP) or a multicopy *amrZ*-containing plasmid (PA14/pUCP-*amrZ*). **(C)** RT-qPCR analysis of *amrZ* mRNA levels in WT PA14 from actively swarming tendril tips, cells localized in the center of swarming colonies, free-swimming planktonic cells, and cells grown as biofilm at 6 h (initial attachment) and 24 h (developed biofilm). The relative gene expression was reported relative to the levels in swarm center cells. Significance was determined by Students’ *t*-test (**p < 0.01).

To evaluate the potential role of AmrZ in the swarming motility of *P. aeruginosa*, we initially constructed an in-frame deletion of *amrZ* in strain PA14 and examined its swarming phenotype. As shown in Figure 1A, deletion of the *amrZ* gene resulted in a severe defect in swarming motility compared to the parental wild-type (WT) strain. The *amrZ* mutant grows at a rate indistinguishable from that of the WT under the same medium conditions (data not shown), suggesting the swarming defect observed was not due to decreased growth rate.

To confirm that the swarming defect of the ∆*amrZ* mutant was indeed due to the loss of *amrZ*, we generated a complementation construct (pTn*7*T-*amrZ*) to express the *amrZ* gene under its own promoter at the chromosomal *att*Tn*7* site (Choi and Schweizer, 2006). As shown in Figure 1A, complementation of the *amrZ* mutant restored the swarming phenotype to WT levels, demonstrating the observed swarming defect is due to the *amrZ* deletion. Moreover, multicopy expression of *amrZ* (pUCP-*amrZ*) in WT PA14 resulted in a hyperswarming phenotype compared to that of the vector control (Figure 1B), reinforcing the finding that AmrZ positively regulates swarming motility of *P. aeruginosa*. In addition, deletion of *amrZ* in the type strain PAO1 also resulted in a strong impairment in swarming motility, and the swarming defective phenotype could be restored to WT levels by native expression of the chromosomally integrated *amrZ*-PAO1 gene (Supplementary Figure S1), suggesting the influence of AmrZ on swarming motility is not strain-specific.

Recent findings suggest that distinct bacterial subpopulations are present within a swarming colony (Tremblay and Deziel, 2010). While cells at the tendril tips are fast moving and rapidly spread on uncolonized surfaces, swarm center populations live in a biofilm-like state allowing a permanent settlement of the colonized areas (Tremblay and Deziel, 2010). To further understand the role of AmrZ in lifestyle adaptation, especially its association with swarming behavior, we analyzed the expression pattern of *amrZ* in swarming colonies of WT PA14 located at the tendril tips, swarming center as well as cells grown planktonically or as biofilms at both 6 h (initial attachment) and 24 h (developed biofilm). It was found that *amrZ* expression in actively swarming tendril tips was upregulated 2.68-fold compared to that in swarming center cells (Figure 1C), and the expression in swarming tips was comparable to that of cells grown planktonically (Figure 1C). The expression of *amrZ* in biofilm cells was downregulated 3.8- and 3.35-fold at 6 and 24 h, respectively, relative to their planktonic counterparts and this decrease was more striking when compared to swarming tendril tip cells (Figure 1C), indicating the expression of *amrZ* in motile cells was higher than that in sessile cells. As loss of *amrZ* leads to a hyper-biofilm phenotype in *P. aeruginosa* (Jones et al., 2013), these results support the notion that AmrZ may serve as a molecular switch that regulates the transition between motile and sessile lifestyles. Collectively, these data suggest that AmrZ is an important contributor to swarming motility, and its expression level is closely correlated with the motile and sessile behaviors of *P. aeruginosa*.

### AmrZ-Mediated Swarming Regulation Does Not Implicate Biosurfactant Production but Is Associated With Flagellar Function

**Figure 2:**
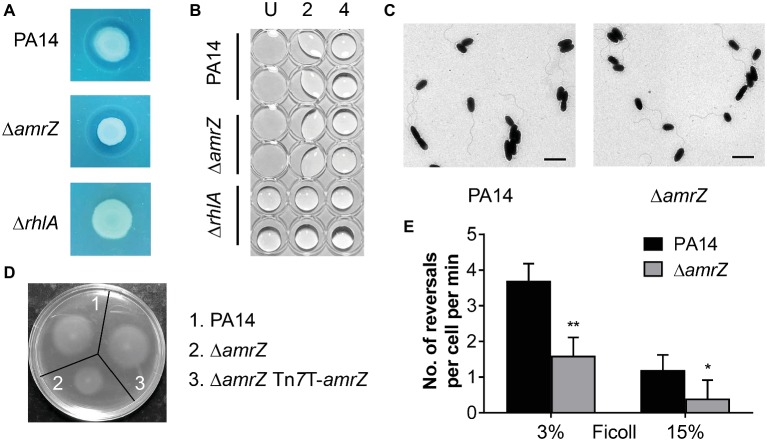
The swarming defect of the *amrZ* mutant is independent of changes of biosurfactant production or flagellar formation but is associated with flagellar malfunction. **(A)** Methylene blue-rhamnolipid plate assay. The presence of surfactant is indicated by the formation of a dark ring surrounding a colony and the non-rhamnolipid-producing *rhlA* mutant served as a negative control. **(B)** Drop collapse analysis of PA14, ∆*amrZ*, and ∆*rhlA* mutants to detect the presence of the rhamnolipid precursor HAA. Samples were diluted in dH_2_O, spotted on the lid of a 96-well plate, and assayed for bead formation. The dilution factors are shown at the top. U, undiluted. **(C)** Morphology of PA14 and *amrZ* mutant cells by TEM of bacteria taken directly from swarm plates. Bar, 2 μm. **(D)** Swimming motility assay of the indicated strains. **(E)** Flagellar reversal rates under low (3% Ficoll) and high-viscosity (15% Ficoll) conditions, representing swimming and swarming conditions, respectively, were measured as changes in direction of movement of cells. Rates are expressed as number of reversals per cell per minute. Error bars represent the standard deviations. *p < 0.05; **p < 0.01 compared with WT by Student’s *t*-test.

Swarming behavior in *P. aeruginosa* is dependent upon a functional flagellum and the production of biosurfactants (mostly rhamnolipids) (Kohler et al., 2000; Jean-Pierre et al., 2016). To examine whether the swarming-deficient phenotype of ∆*amrZ* was related to a lack of biosurfactant synthesis, rhamnolipid production assays were first performed (Caiazza et al., 2005; Yeung et al., 2009). The *rhlA* mutant was included as a control since this mutant has been shown to be defective in rhamnolipid production (Deziel et al., 2003; Caiazza et al., 2005). We found that the *amrZ* mutant had a dark blue halo around the colony similar to that for the WT (Figure 2A), while the *rhlA* mutant had no visible haloes around the colony, indicating that the *amrZ* mutant was not defective in the production of rhamnolipid. We also measured the production of rhamnolipid precursor 3-(3-hydroxyalkanoyloxy)alkanoic acids (HAAs) by a drop collapse assay (Caiazza et al., 2005; Murray and Kazmierczak, 2008), and found the ∆*amrZ* strain showed equivalent drop collapse activity compared to the WT (Figure 2B). Thus, the defect in Δ*amrZ* mutant swarming is likely not due to a deficiency in HAA and/or rhamnolipid production.

Another essential factor that contributes to swarming of *P. aeruginosa* is flagellum biosynthesis or function. To better understand how AmrZ affects swarming, TEM studies were carried out to determine whether the changes in swarming behavior were due to changes in biosynthesis of the flagellar apparatus. As previously noted for strain PAO1 (Baynham et al., 2006), the *amrZ* mutant in PA14 possessed well-developed flagella and the percentage of flagellated cells was comparable to that of the WT (88 ± 4% for WT strain and 85 ± 6% for *amrZ* mutant) (Figure 2C), indicating that there was no defect in flagella formation. We next tested whether flagellar functionality was responsible for the ∆*amrZ* swarming phenotype by performing swimming motility assays, since swimming motility depends entirely on a functional flagellum (Yeung et al., 2009). As seen in Figure 2D, the ∆*amrZ* mutant had a severe defect in swimming motility and could be restored by complementation with a functional copy of *amrZ*, indicating that AmrZ is necessary for flagellar functioning. Furthermore, we examined the effect of AmrZ on flagellar reversal rates in liquid media with viscosities mimicking swimming (3% Ficoll) and swarming (15% Ficoll) conditions (Caiazza et al., 2007). In accord with motility assays on agar plates, inactivation of *amrZ* resulted in significantly reduced frequency of flagellar reversals under both conditions (Figure 2E), suggesting that AmrZ is involved in flagellar activity. Taken together, these results demonstrate that the swarming repression in the ∆*amrZ* mutant occurs *via* flagellar malfunction rather than a default in flagellum biosynthesis or biosurfactant production.

### Increased Levels of Cyclic di-GMP Is Partially Responsible for the Swarming Defect of the ∆*amrZ* Mutant

**Figure 3:**
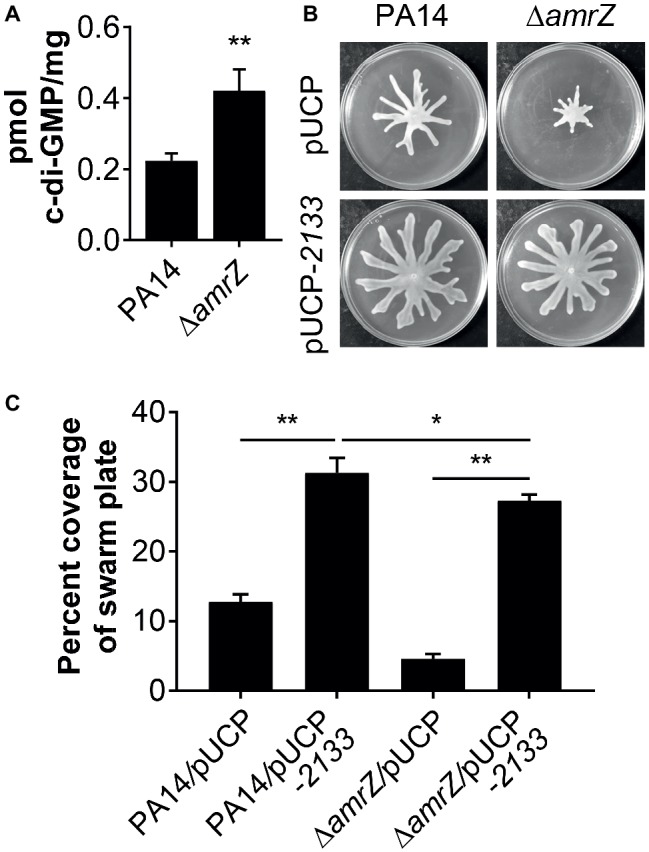
c-di-GMP contributes to the swarming defects of the *amrZ* mutant. **(A)** Measurements of c-di-GMP by LC-MS/MS for the indicated strains grown on swarm plates. Data are expressed as pmol of c-di-GMP/mg of dry weight of the cell pellets from which the nucleotides were extracted. **(B)** Representative swarming images of the WT or *amrZ* mutant carrying either the empty vector or pUCP-*2133* for overproducing the PA2133 phosphodiesterase as indicated. **(C)** Percent coverage of swarm plates for the strains shown in **(B)**. Statistical analysis is based on three replicates and significance was determined with Student’s *t*-test (*p < 0.05; **p < 0.01).

It has been previously demonstrated that a ∆*amrZ* mutant exhibited elevated levels of c-di-GMP over that of the parental PAO1 strain (Jones et al., 2014), and it could be expected that c-di-GMP levels will also be higher in a PA14 ∆*amrZ* mutant strain. However, it was unknown if this would also be the case in swarming cells and whether c-di-GMP was involved in the repression of swarming in ∆*amrZ*. Therefore, we compared the intracellular levels of c-di-GMP between WT PA14 and the ∆*amrZ* mutant under swarming conditions. As shown in Figure 3A, c-di-GMP levels were about two times higher in the *amrZ* mutant than in the WT. This observation is consistent with the previous report by Jones and colleagues (Jones et al., 2014) and coincides with the established notion that high c-di-GMP levels repress swarming motility. To further investigate whether the elevated level of c-di-GMP was responsible for the swarming defect of the ∆*amrZ* mutant, PA14 and the ∆*amrZ* mutant strains were transformed with a plasmid encoding the PDE PA2133 (pUCP-*2133*), which has previously been used to artificially reduce c-di-GMP levels in *P. aeruginosa* (Moscoso et al., 2011; Frangipani et al., 2014; Li et al., 2017). We found that overexpression of PA2133 led to a significant increase of swarming motility in both WT and ∆*amrZ* mutant strains (Figures 3B,C). Additionally, we confirmed that WT and ∆*amrZ* mutant cells overexpressing PA2133 had very low to undetectable levels of c-di-GMP (data not shown). However, as the absence of *amrZ* still has an effect on swarming even when the PDE is overexpressed, these data suggest that c-di-GMP could be involved in the repression of swarming motility in the ∆*amrZ* mutant.

### The GcbA-Derived Cyclic di-GMP Is Involved in the Swarming Repression Observed in the Δ*amrZ* Mutant

**Figure 4:**
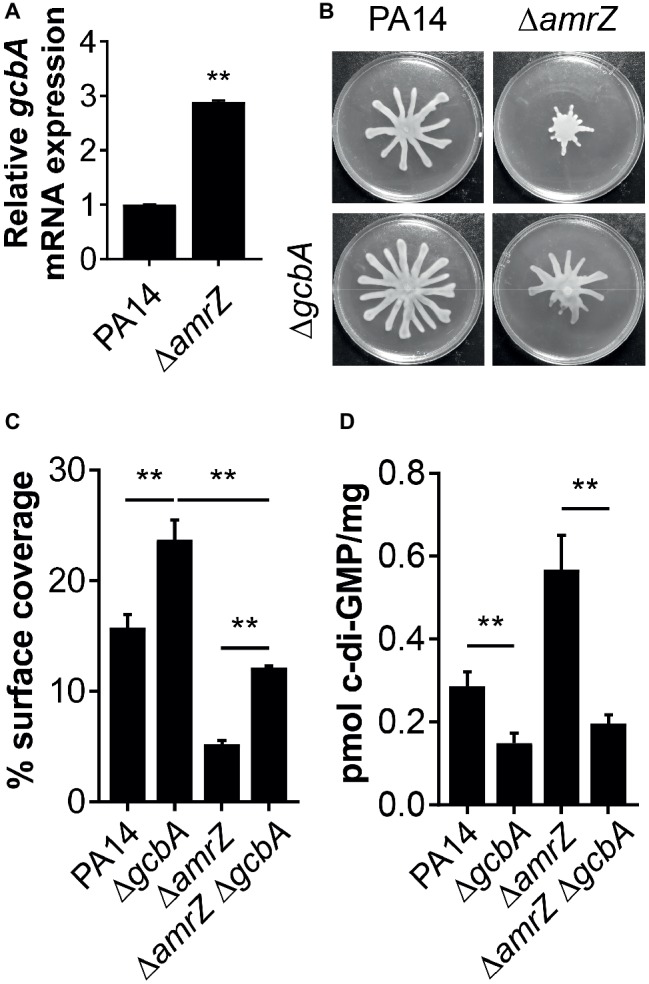
GcbA is required for increased c-di-GMP accumulation in Δ*amrZ* and partially responsible for its swarming phenotype. **(A)** Relative mRNA levels of *gcbA* in indicated strains under swarming conditions as determined by real-time PCR. **(B)** Representative images of swarming motilities of indicated strains. **(C)** Percent coverage of swarm plates by the respective swarms. **(D)** Quantification of c-di-GMP levels of the indicated strains grown on swarm plates. **p < 0.01 as determined by Student’s *t*-test.

Our data suggest that there is a c-di-GMP-dependent control of swarming motility in the ∆*amrZ* mutant. However, as multiple c-di-GMP-metabolizing enzymes exist in *P. aeruginosa*, we are interested in which DGCs or PDEs could be responsible for the changes in the internal c-di-GMP pool in ∆*amrZ* and hence swarming motility. In light of the finding that the mRNA expression of the DGC GcbA (also named AdcA) displayed the highest level of upregulation in a transcriptional profiling analysis of a PAO1 *amrZ* mutant (Jones et al., 2014), we asked whether GcbA plays a role in modulating the c-di-GMP concentration to mediate repression of swarming in ∆*amrZ*. For the purpose of this study, we validated the expression of *gcbA* under swarming conditions by qRT-PCR and found the *gcbA* gene was significantly upregulated (2.89-fold) in ∆*amrZ* compared to the parental PA14 strain (Figure 4A); however, this upregulation was smaller than that observed in PAO1 ∆*amrZ* (40.27-fold by RNA-Seq) (Jones et al., 2014). While the RNA-Seq was performed under liquid LBNS (LB medium with no salt) conditions, this result suggests that the viscosity or composition of the environment may cause AmrZ to exert different degrees of downstream effects. We next engineered a ∆*amrZ* ∆*gcbA* double mutant and examined its swarming phenotype as well as the levels of c-di-GMP. As a control, a *gcbA* single mutant was also included. We observed that swarming motility was largely restored in the ∆*amrZ* ∆*gcbA* mutant compared to that of the ∆*amrZ* strain, and the ∆*gcbA* single mutant also exhibited significantly enhanced swarming relative to the WT (Figures 4B,C). Analysis of the c-di-GMP levels showed that the ∆*amrZ* ∆*gcbA* double mutant exhibited significantly reduced c-di-GMP levels compared to that in the ∆*amrZ* mutant (Figure 4D). However, it is noteworthy that while the ∆*amrZ* ∆*gcbA* mutant had a c-di-GMP content at almost the levels of the Δ*gcbA* single mutant (Figure 4D), swarming motility in the Δ*amrZ* Δ*gcbA* mutant was still 2-fold lower than that of the Δ*gcbA* mutant (Figure 4C); this result implies that AmrZ also affects swarming motility through a *gcbA*- and (likely) c-di-GMP-independent mechanism. Together these data suggest that GcbA is responsible for the increased production of c-di-GMP in ∆*amrZ* and the swarming deficiency phenotype of the ∆*amrZ* mutant is, at least partially, dependent on GcbA-synthesized c-di-GMP.

### FlgZ and PA14_56180 Are the Effector Relay Proteins That Respond to GcbA Cyclic di-GMP Signaling to Mediate Repression of Swarming Motility

**Figure 5:**
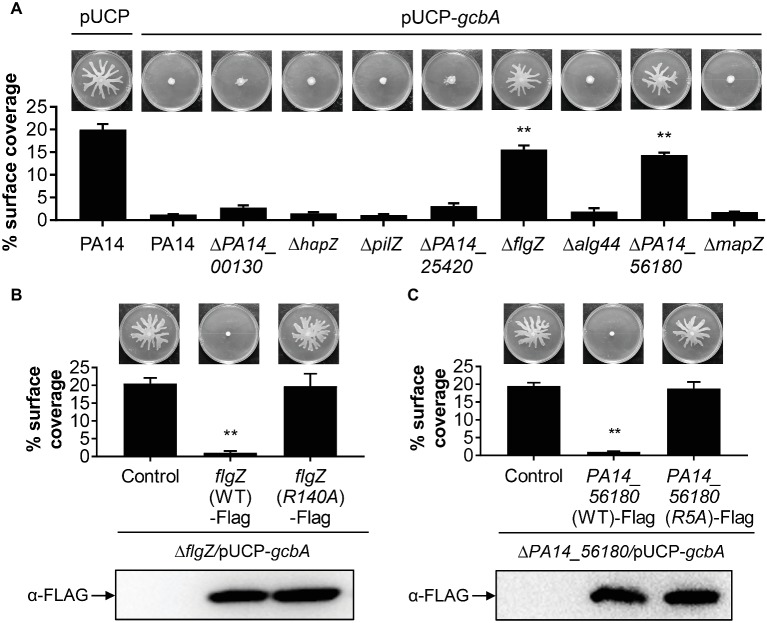
FlgZ and PA14_56180 are required for GcbA c-di-GMP-mediated swarming repression. **(A)** Swarming phenotype and quantification of the percentages of plate surface coverage of the indicated strains carrying either the empty vector (pUCP) or the GcbA overproducing plasmid pUCP-*gcbA*. **p < 0.01 (Student’s *t*-test, compared to PA14/pUCP-*gcbA*). **(B)** Representative swarming plates (upper row) and average percentage of the plate coverage (middle row) by Δ*flgZ*/pUCP-*gcbA* carrying the chromosomally encoded C-terminally FLAG-tagged WT FlgZ or FlgZ (R140A) under the control of the P*_BAD_* promoter at the *att*Tn*7* site. Significance was determined by Student’s *t*-test (**p < 0.01) relative to the vector control. Lower row: Western analysis of the FLAG-tagged FlgZ derivatives in indicated strains. Samples from equivalent number of bacterial cells were loaded onto SDS-PAGE gels and probed with anti-FLAG antibody. **(C)** Swarm analysis (upper row), quantification of percent coverage of swarm plates (middle row), and protein expression levels of FLAG-tagged derivatives of PA14_56180 (lower row) in Δ*PA14_56180*/pUCP-*gcbA* harboring the chromosomally encoded C-terminally FLAG-tagged WT PA14_56180 or PA14_56180 (R5A) as under panel **(B)**.

We next set out to identify the downstream target(s) of GcbA c-di-GMP signaling, and expected certain c-di-GMP-binding effectors would likely be involved in this response. The *P. aeruginosa* genome encodes eight PilZ domain proteins, and seven of them are able to bind to c-di-GMP (Merighi et al., 2007). The seven PilZ domain proteins include HapZ (PA14_27930), FlgZ (PA14_20700), Alg44 (PA14_18550), and MapZ (PA14_60970) with known physiological roles and three other proteins (PA14_00130, PA14_25420, and PA14_56180) with unknown function. The PilZ (PA14_25770) shows no detectable binding of c-di-GMP in an *in vitro* assay but may interact with c-di-GMP *in vivo* (Merighi et al., 2007). To investigate whether PilZ domain proteins participate in the GcbA c-di-GMP mediated repression of swarming, we constructed deletions of each of the PilZ domain protein-encoding gene in the WT background, and the *gcbA* gene carried on a multi-copy plasmid (pUCP-*gcbA*) was introduced into these strains. The data revealed that overexpression of *gcbA* in WT PA14 completely abolished swarming motility compared with the vector control (Figure 5A, first two panels). Furthermore, overexpression of *gcbA* in most of the mutants lacking PilZ-domain bearing proteins, repressed swarming. The exceptions were the *flgZ* and *PA14_56180* mutant strains. This strongly suggests that these genes are genetically linked to *gcbA* in controlling swarming motility in *P. aeruginosa*.

PilZ domain proteins bind c-di-GMP by the conserved motifs RXXXR and (D/N)XSXXG (Cheang et al., 2019). Sequence alignment suggests that FlgZ and PA14_56180 contain both of these motifs (Supplementary Figure S2). In *Pseudomonas putida*, amino acid substitution analysis of FlgZ showed that alanine substitution of the first arginine in the RXXXR motif results in complete loss of c-di-GMP binding (Ko et al., 2010). Similarly, a corresponding replacement (R140A) of *P. aeruginosa* FlgZ abolishes the capacity of FlgZ to respond to c-di-GMP (Baker et al., 2016). Sequence alignment with orthologs from other species suggests that PA14_56180 is likely to use the same set of key residues (Supplementary Figure S2) for c-di-GMP binding (Xu et al., 2016a). To further validate whether c-di-GMP binding is required for FlgZ or PA14_56180 to mediate GcbA regulation of swarming, we generated C-terminally FLAG-tagged FlgZ-R140A and PA14_56180-R5A derivatives carrying amino acid substitution of the first arginine residue (R) in the RXXXR motif and assessed whether these mutant proteins were functional in swarming repression. As shown in Figures 5B,C, neither of these mutant variants mediated a reduced swarming phenotype in the GcbA overproducing background, even though they were expressed at equivalent levels as the WT protein, which readily repressed swarming in this background (Figures 5B,C). These data suggest that the conserved residue required for c-di-GMP binding is critical for FlgZ and PA14_56180 to mediate swarming repression in response to GcbA c-di-GMP signaling.

Furthermore, we asked whether expression of *flgZ* and *PA14_56180* was affected by GcbA under the conditions studied here. qRT-PCR revealed that no significant differences in the transcript abundance of *flgZ* (fold change, 0.95 ± 0.05, for Δ*gcbA* versus PA14) or *PA14_56180* (fold change, 0.83 ± 0.04) was noted between the Δ*gcbA* mutant and WT PA14 strain. Overexpression of *gcbA* similarly had no effect on expression of *flgZ* (fold change, 0.89 ± 0.03, for PA14/pUCP-*gcbA* versus PA14/pUCP) or *PA14_56180* (fold change, 1.08 ± 0.06), suggesting that GcbA does not affect the transcription of *flgZ* and *PA14_56180*. Collectively, these results indicate that FlgZ and PA14_56180 function as the GcbA c-di-GMP effectors to mediate swarming repression in *P. aeruginosa*.

### AmrZ Regulates Swarming Motility Through the GcbA-FlgZ/PA14_56180 Signal Transduction Pathway

**Figure 6:**
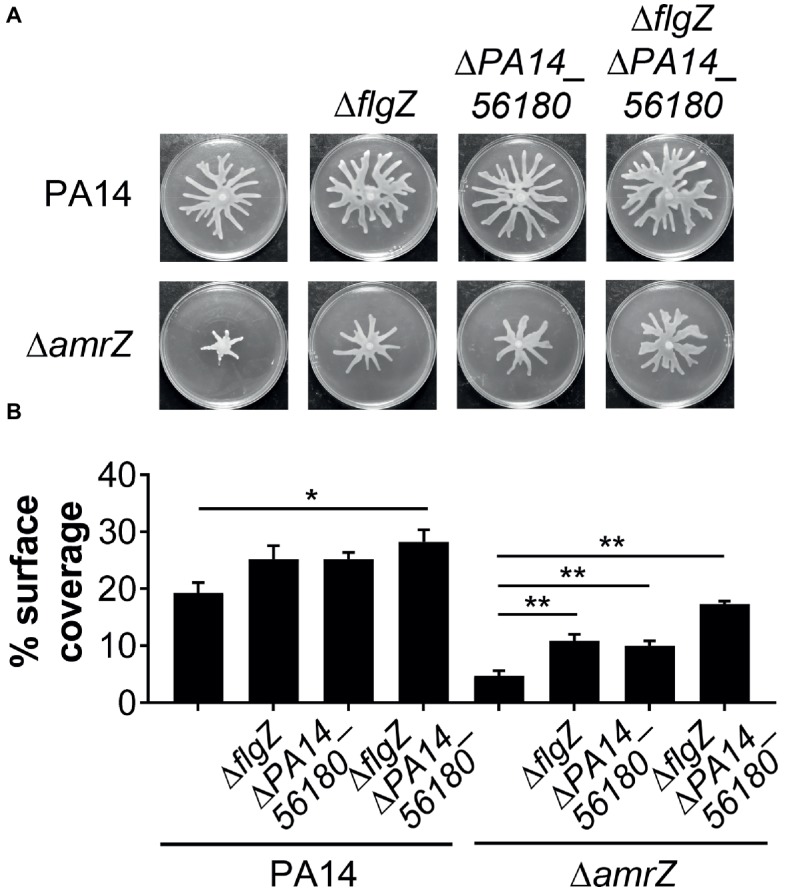
FlgZ and PA14_56180 contribute to swarming motility repression of the *amrZ* mutant. **(A)** Representative swarm plates of the indicated strains. **(B)** Percentage of the plate surface occupied by the respective swarms shown in **(A)**. Significance was determined using one-way ANOVA followed by Tukey’s multiple comparison test (*p < 0.05; **p < 0.01).

Since GcbA contributes to the swarming repression in Δ*amrZ*, and FlgZ and PA14_56180 participate in the GcbA-mediated repression of swarming, we reasoned that if the negative regulation of swarming by GcbA occurred through the FlgZ/PA14_56180 pathway in Δ*amrZ*, deleting *flgZ* or *PA14_56180* would be able to restore swarming to the Δ*amrZ* mutant. Therefore, we constructed mutations in either *flgZ* or *PA14_56180* or both in the Δ*amrZ* mutant background as well as in WT as a control. As shown in Figures 6A,B, while deletion of either of the *flgZ* and *PA14_56180* genes in Δ*amrZ* resulted in significantly enhanced motility compared to the Δ*amrZ* mutant, the Δ*amrZ* Δ*flgZ* Δ*PA14_56180* triple mutant showed a more pronounced swarming phenotype than the individual Δ*amrZ* Δ*flgZ* or Δ*amrZ* Δ*PA14_56180* mutants. However, the Δ*amrZ* Δ*flgZ* Δ*PA14_56180* triple mutant still had a weaker swarming phenotype than the Δ*flgZ* Δ*PA14_56180* double mutant, indicating that other factors are also involved in AmrZ-mediated swarming repression. Furthermore, we examined the expression of the two genes in Δ*amrZ* by qRT-PCR. Consistent with the previous RNA-Seq reports in PAO1 (Jones et al., 2014), we found the transcript abundances of *flgZ* and *PA14_56180* in Δ*amrZ* were significantly increased (2.23 ± 0.06 and 2.42 ± 0.04, respectively) relative to the WT strain; together with the increased transcript levels of *gcbA* in Δ*amrZ* (Figure 4A), these results suggest a coordinated transcriptional regulation of *gcbA*, *flgZ* and *PA14_56180* by AmrZ. Overall, these data suggest that swarming motility inhibition in the *amrZ* mutant is in part controlled by a c-di-GMP signaling module that consists of the DGC GcbA and two c-di-GMP receptors containing PilZ domains: FlgZ and PA14_56180.

### Pel Exopolysaccharide Acts Additively With GcbA for Swarming Regulation in Δ*amrZ*

**Figure 7:**
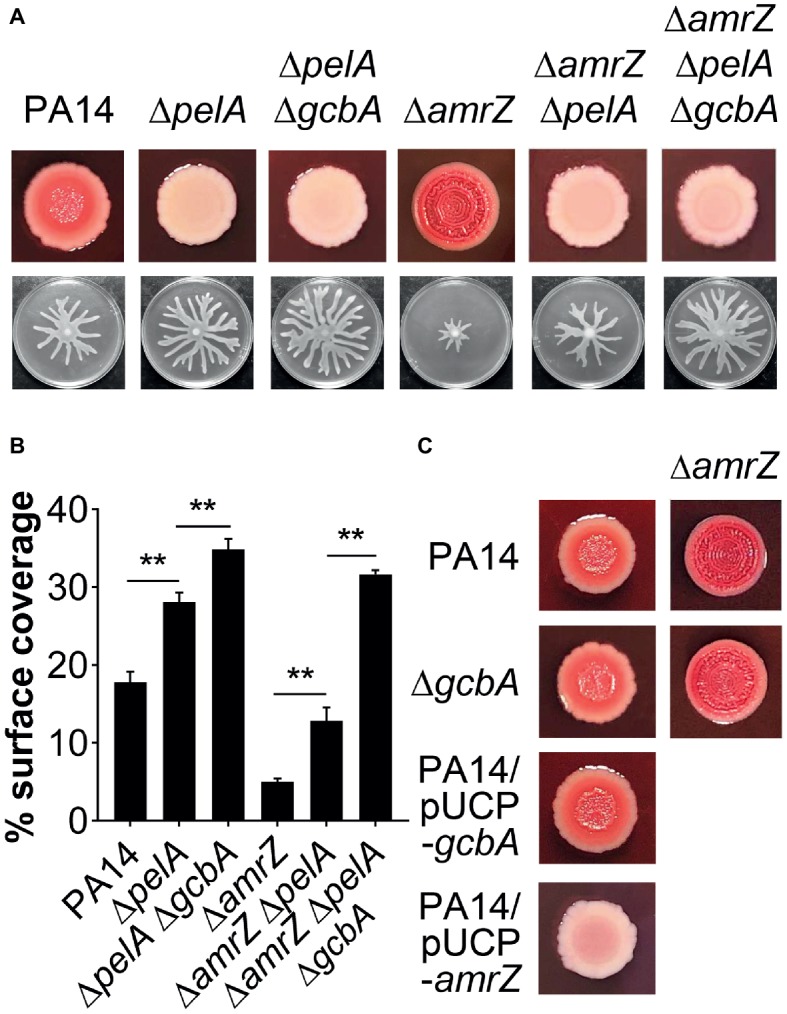
The *pel*-derived polysaccharide collaborates with GcbA to contribute to the swarming defect of Δ*amrZ*. **(A)** CR binding (upper row) and representative swarm plates (lower row) of the indicated strains. **(B)** Quantification of the extent of swarming for the strains indicated in **(A)**. **(C)** CR-binding phenotypes of the indicated strains. The name of the corresponding strain is indicated on the left. In PA14 and Δ*gcbA* additional *amrZ* mutation was introduced as indicated at the top of the second column. Significance was determined by one-way ANOVA followed by Tukey’s multiple comparison test (**p < 0.01).

The epistasis studies with the *amrZ* and *gcbA*/*flgZ*/*PA14_56180* genes described above indicated that eliminating GcbA signaling pathway in the Δ*amrZ* mutant background results in a significant, but not completely restoration of swarming motility (Figures 4B,C, 6A,B). Thus, there should be some other factor(s) responsible for the swarming defect in the Δ*amrZ* mutant. In PA14, exopolysaccharide (EPS) has been shown to be a regulator of swarming motility (Caiazza et al., 2007). We noticed that the *amrZ* mutant displayed an aggregated colony morphology when grown on agar plates, which is usually linked to overproduction of EPS (Kirisits et al., 2005). When tested on Congo red (CR) plates, the *amrZ* mutant indeed showed hyper-binding to CR and displayed a more wrinkly phenotype compared to the WT (Figure 7A; Supplementary Figure S3A), suggesting the *amrZ* mutant produced substantially more polysaccharides. The primary polysaccharide in PA14 is synthesized by the *pel* operon and this is the cellular component bound by CR (Friedman and Kolter, 2004; Lee et al., 2007). To investigate whether enhanced polysaccharide production contributes to the swarming repression in Δ*amrZ*, we introduced a *pelA* mutation into the Δ*amrZ* strain and examined its EPS production and swarming phenotype. The results showed that the *pelA* mutation eliminates both the CR binding and the wrinkled phenotype of the *amrZ* mutant (Figure 7A; Supplementary Figure S3A), and swarming motility was largely restored in the Δ*amrZ* Δ*pelA* mutant (Figures 7A,B), indicating that overproduction of Pel polysaccharide is responsible for the swarming defect observed in the *amrZ* mutant.

Regulation of Pel expression is complex. To assess whether increased Pel polysaccharide production in Δ*amrZ* is due to overexpression of the *pel* genes, we measured the transcript levels of the first (*pelA*) and last gene (*pelG*) of the *pel* operon (Friedman and Kolter, 2004) using qRT-PCR. Interestingly, we found that in cells grown on swarming plates, there was no significant change in *pelA* (fold change, 1.09 ± 0.04, for Δ*amrZ* versus PA14) or *pelG* (fold change, 1.15 ± 0.01) expression between the WT and Δ*amrZ* mutant. Similar results were observed when these strains were grown on CR plates under conditions identical to those used for CR assays (data not shown). These data suggest that the impact of AmrZ on Pel polysaccharide production in PA14 occurs *via* a nontranscriptional mechanism.

To determine whether GcbA c-di-GMP signaling crosstalks to the EPS production in Δ*amrZ*, we tested the CR binding ability of the Δ*amrZ* Δ*gcbA* mutant with the Δ*amrZ* strain. Interestingly, we observed that the Δ*amrZ* Δ*gcbA* mutant exhibited a CR binding phenotype comparable to that of the Δ*amrZ* mutant (Figure 7C; Supplementary Figure S3B). As a control, inactivation or overexpression of *gcbA* in WT PA14 also had no effect on CR binding (Figure 7C; Supplementary Figure S3B), which is consistent with a previous report that GcbA does not affect the transcription of *pel* genes (Petrova et al., 2014). Thus, these findings indicate that AmrZ regulation of EPS production is independent of GcbA c-di-GMP signaling. Consistent with this, inactivation of *gcbA* in the Δ*amrZ* Δ*pelA* mutant revealed additive effects on restoring the swarming defect of the *amrZ* mutant (Figures 7A,B), and the double Δ*pelA* Δ*gcbA* mutant also exhibited a significant increase of swarming relative to that observed for the Δ*pelA* mutant (Figures 7A,B). Together, these data suggest that Pel polysaccharide and GcbA act in concert to contribute to the repression of swarming in Δ*amrZ*.

## Discussion

During colonization and infection, pathogenic bacteria such as *P. aeruginosa* has evolved several mechanisms to rapidly adjust gene expression to enable appropriate physiological and molecular adaptations. The transcription factor AmrZ is one of these regulators that has been implicated in the controlling of multiple cellular processes associated with virulence and environmental fitness (Jones et al., 2014; Martinez-Granero et al., 2014). Here, we are adding an important piece to the AmrZ regulon by showing that AmrZ is an important contributor to the regulation of surface-based swarming motility in *P. aeruginosa*. We have also demonstrated that the shifts in *amrZ* gene expression is associated with lifestyle changes, wherein actively moving cells express higher relative level of *amrZ* while a downregulation is observed in the biofilm growth mode. Combined with the fact that inactivation of *amrZ* results in enhanced level of biofilm formation (Jones et al., 2013), our results highlight the concept that AmrZ may serve as a molecular switch that controls the transition between motile and sessile lifestyles.

In agreement with the role of AmrZ in positive control of flagellum-driven motility in this study, recent findings have suggested that AmrZ functions as a positive regulator of motility in *Pseudomonas syringae* (Prada-Ramirez et al., 2016) and *Pseudomonas stutzeri* (Baltrus et al., 2018). In *P. syringae*, AmrZ activates the expression of the flagellin gene *fliC* and the *amrZ* mutant produces less flagella than the parental strain (Prada-Ramirez et al., 2016). This is in contrast to *P. aeruginosa*, where inactivation of *amrZ* does not affect the flagellin production (Baynham et al., 2006) or flagellation on the cell surface (Figure 2C). In *P. stutzeri*, AmrZ has also been shown to positively regulate colony spreading, a type of bacterial motility that is phenotypically presumed to be correlated with swarming motility (Baltrus et al., 2018). Interestingly, the *P. aeruginosa* AmrZ is able to complement both the swimming and colony spreading defects of the *P. stutzeri amrZ* mutant (Baltrus et al., 2018), suggesting that the two versions of this protein likely regulate motility in a similar way. However, the molecular mechanisms of motility regulation by AmrZ in *P. stutzeri* have not been determined.

Our work reveals that the second messenger c-di-GMP produced by the DGC GcbA is involved in the AmrZ-mediated regulation of swarming. c-di-GMP has long been known as a regulatory maestro that is involved in regulation of many cellular processes, including flagellum-based motility (Wolfe and Visick, 2008). GcbA was previously found to be an enzymatically active DGC participating in the regulation of motility in *Pseudomonas fluorescens* (Newell et al., 2011) and *P. aeruginosa* (Petrova et al., 2014). While the *P. fluorescens* GcbA inhibits swimming motility by a yet undetermined mechanism (Newell et al., 2011), *P. aeruginosa* GcbA was found to negatively regulate flagellum-driven motility by suppressing flagellar reversal rates (Petrova et al., 2014). Furthermore, the regulation of motility by GcbA is partially dependent on a small RNA (sRNA) RsmZ, and GcbA levels were found to positively correlate with the abundance of RsmZ (Petrova et al., 2014). In *P. aeruginosa*, transcription of *rsmZ* is under the direct control of the GacS/GacA two-component system (Brencic et al., 2009). However, there is no evidence of AmrZ regulating any of the members of the Gac/Rsm signaling cascade (Jones et al., 2014), and thus, other regulatory elements associated with GcbA c-di-GMP signaling might come into action to regulate motility in Δ*amrZ*.

In the past decades, the mechanisms of c-di-GMP turnover have been extensively studied (Schirmer and Jenal, 2009), yet the signal transduction mechanism such as the receptor(s) involved in specific DGCs/PDEs-associated c-di-GMP sensing still remain largely unknown (Krasteva et al., 2012). In this study, we provide evidence suggesting that two PilZ domain proteins FlgZ and PA14_56180 directly sense GcbA c-di-GMP signaling and act in concert to contribute to the repression of swarming motility in Δ*amrZ*. The *flgZ* gene is located downstream of the *flgMN* genes in a flagellar operon and encodes a homologue to YcgR (Baker et al., 2016), a c-di-GMP-binding protein in *E. coli* and *Salmonella* that acts as a flagellar brake to mediate c-di-GMP responsive control of motility (Paul et al., 2010). In *P. aeruginosa*, Baker et al. demonstrated that FlgZ interacts specifically with the flagellar stator protein MotC in a c-di-GMP-dependent manner, and absence of FlgZ suppressed the effect of the absence of a PDE (*bifA*) with regards to repression of swarming (Baker et al., 2016). Here, we found that FlgZ also contributes to GcbA c-di-GMP-mediated swarming repression (Figure 5), making GcbA a new DGC associated with the c-di-GMP receptor FlgZ. In addition, our study also identified PA14_56180 as being required for GcbA-mediated repression of swarming motility (Figure 5). Nevertheless, Baker and colleagues did not find PA14_56180, but only FlgZ as a regulator of swarming in the response to elevated c-di-GMP levels due to the absence of *bifA* (Baker et al., 2016). This suggests that PA14_56180 may specifically respond to GcbA c-di-GMP signaling while FlgZ may respond to a global pool of c-di-GMP. However, to understand the specificity and generality of these receptors in c-di-GMP signal transmission will require the identification of individual c-di-GMP signaling pathway of the multiple DGCs and PDEs encoded in *P. aeruginosa* genome. In another study, Lewis and colleagues performed a screen for proteins involved in c-di-GMP-mediated response to ethanol which led to the identification of a few candidates including GcbA and FlgZ (Lewis et al., 2019); however, the relationship between the two proteins was not investigated in this research, and our findings may provide an explanation for this screening result. Unlike FlgZ, there is limited information available about PA14_56180, but our results highlight a role for PA14_56180 as a GcbA c-di-GMP receptor affecting swarming motility of *P. aeruginosa*. Besides, the expression of *gcbA*, *flgZ* and *PA14_56180* are all upregulated in the *amrZ* mutant, suggesting that these genes are cotranscriptionally regulated. Furthermore, the participation of PA14_56180 in the GcbA pathway may hint at potential for colocalization of GcbA and PA14_56180 within the cell. However, it is also possible that other c-di-GMP signaling could feed in to modulate swarming motility through PA14_56180. Future studies will be required to determine PA14_56180 subcellular localization and the molecular basis for downstream signaling, including the impact of c-di-GMP binding on the structure and function of PA14_56180. Elucidating such questions will substantially enhance our understanding of c-di-GMP signaling mechanisms.

In addition to the GcbA-FlgZ/PA14_56180 signal transduction cascade, our data suggest that AmrZ can modulate another facet of swarming by coordinating the production of Pel polysaccharide. We show that the *amrZ* mutant produces more EPS and overexpression of *amrZ* in WT leads to repression of EPS production (Figures 7A,C; Supplementary Figure S3B). However, the Δ*amrZ* mutation does not alter *pel* mRNA expression, suggesting that AmrZ impacts Pel polysaccharide *via* a nontranscriptional mechanism. This is distinct from the RNA-seq data in PAO1, where AmrZ activates expression of the *pel* polysaccharide operon (Jones et al., 2014). We also confirmed that a slight decrease in CR binding was observed for a PAO1 *amrZ* mutant (RCRB value: 0.07 ± 0.01) compared to that of the WT PAO1 (0.12 ± 0.01). Indeed, PAO1 differs from PA14 in that PAO1 uses Psl as the primary matrix polysaccharide, while PA14 has a three-gene deletion in the *psl* operon and uses Pel (Colvin et al., 2012; Visaggio et al., 2015). One possible explanation for this is the differences in experimental conditions where some other regulatory elements might come into action to affect the function of AmrZ in transcriptional regulation, or some yet-to-be discovered post-transcriptional mechanisms maybe involved in the AmrZ-mediated regulation of Pel production in PA14. A possible candidate for this post-transcriptional regulation is the AlgC protein which is a key enzyme that provides sugar precursors for the synthesis of *P. aeruginosa* exopolysaccharides including Pel (Ma et al., 2012). Whether the expression or activity of AlgC is altered in Δ*amrZ* and the role of AlgC in regulation of the synthesis of Pel polysaccharide in PA14 will be the subject of future studies. Furthermore, our results suggest that the Pel polysaccharide acts in concert with GcbA signal to contribute to the swarming repression in Δ*amrZ*, as combinatorial analysis of them revealed additive effects on swarming. Similar collaboration has also been found in *Salmonella*, whereby the EPS cellulose works in cooperation with the c-di-GMP-binding protein YcgR to inhibit flagellar motility under high c-di-GMP conditions (Zorraquino et al., 2013). In the *P. aeruginosa* Δ*bifA* and Δ*hptB* mutants that exhibit elevated levels of c-di-GMP, Pel polysaccharide and FlgZ also function together to mediate c-di-GMP-dependent repression of swarming (Baker et al., 2016). However, including the AmrZ-dependent mechanism reported here, we cannot rule out the possibility that Pel polysaccharide and the downstream mediators (such as FlgZ, PA14_56180, etc.) of the c-di-GMP signaling pathway may influence each other. This issue will be elucidated in future studies.

**Figure 8:**
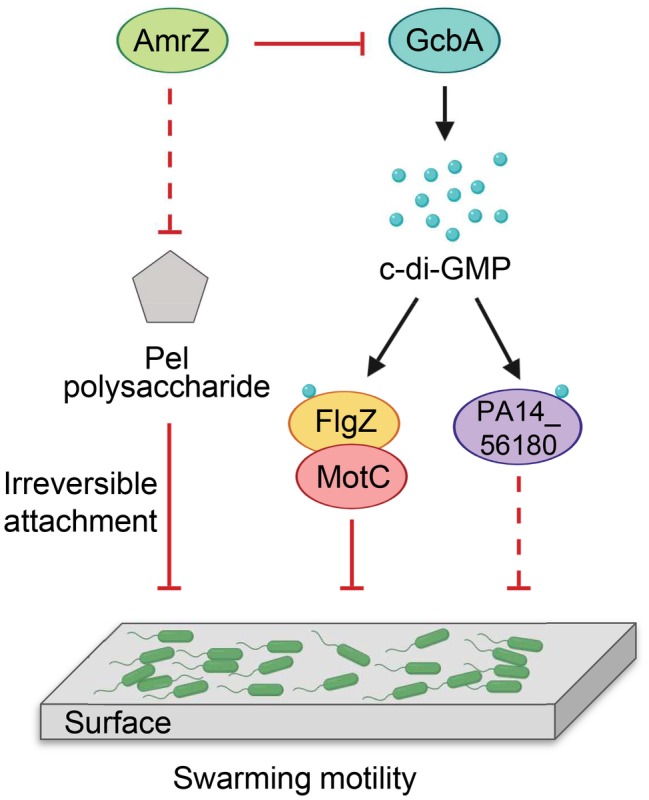
Model for AmrZ-mediated swarming motility control. AmrZ affects swarming by negatively regulating the production of Pel polysaccharide and a GcbA-dependent c-di-GMP signaling. The Pel polysaccharide inhibits swarming motility by promoting the transition from reversible to irreversible attachment (Caiazza et al., 2007). The GcbA c-di-GMP signal can be further sensed by two PilZ-containing proteins, FlgZ and PA14_56180. Binding of c-di-GMP to FlgZ induces its interaction with the MotC stator and impairs flagellar function probably by preventing the engagement of MotCD with the rotor (Baker et al., 2016). PA14_56180 regulates swarming in a c-di-GMP-dependent manner, but the downstream target(s) of PA14_56180 remains unknown. The solid lines represent direct regulations, and the dashed lines indicate probable indirect regulations.

Our findings are summarized in a model shown in Figure 8. We propose that *P. aeruginosa* adjusts the expression of *amrZ* in response to proper environmental signals such as nutritions or changes in medium viscosity during surface growth. Through AmrZ, *P. aeruginosa* could modulate swarming motility by negatively regulating Pel polysaccharide production and a GcbA-dependent c-di-GMP signaling. While the Pel polysaccharide contributes to the progression toward irreversible attachment (Caiazza et al., 2007), the GcbA c-di-GMP signal is then transmitted *via* FlgZ to the flagellar stator MotC, resulting in impairment of flagellar function (Baker et al., 2016). The GcbA signal can also be sensed by another c-di-GMP receptor PA14_56180, which impacts swarming through an as-yet-unknown mechanism. In view of the importance of bacterial motility for their survival in a natural environment and during infection inside a host, with such a double checkpoint mechanism, AmrZ would allow *P. aeruginosa* to delicately control its surface-associated behaviors, thus enabling better adaptations in response to environmental fluctuations.

## Data Availability

All datasets generated for this study are included in the manuscript and/or the Supplementary Files.

## Author Contributions

KL, QB, and LH conceived and designed the experiments. LH, AD, and QC performed the experiments. LH, KL, and QB analyzed the data. LH and KL wrote the paper.

### Conflict of Interest Statement

The authors declare that the research was conducted in the absence of any commercial or financial relationships that could be construed as a potential conflict of interest.

## References

[ref1] Allsopp L. P. Wood T. E. Howard S. A. Maggiorelli F. Nolan L. M. Wettstadt S.. (2017). RsmA and AmrZ orchestrate the assembly of all three type VI secretion systems in *Pseudomonas aeruginosa*. Proc. Natl. Acad. Sci. USA 114, 7707–7712. 10.1073/pnas.1700286114, PMID: 28673999 PMC5530658

[ref2] Amikam D. Galperin M. Y. (2006). PilZ domain is part of the bacterial c-di-GMP binding protein. Bioinformatics 22, 3–6. 10.1093/bioinformatics/bti739, PMID: 16249258

[ref3] Andrews G. P. Maurelli A. T. (1992). *mxiA* of *Shigella flexneri* 2a, which facilitates export of invasion plasmid antigens, encodes a homolog of the low-calcium-response protein, LcrD, of *Yersinia pestis*. Infect. Immun. 60, 3287–3295. 10.1007/BF01989988, PMID: 1639496 PMC257313

[ref4] Baker A. E. Diepold A. Kuchma S. L. Scott J. E. Ha D. G. Orazi G.. (2016). PilZ domain protein FlgZ mediates cyclic di-GMP-dependent swarming motility control in *Pseudomonas aeruginosa*. J. Bacteriol. 198, 1837–1846. 10.1128/JB.00196-16, PMID: 27114465 PMC4907108

[ref5] Baker A. E. Webster S. S. Diepold A. Kuchma S. L. Bordeleau E. Armitage J. P.. (2019). Flagellar stators stimulate c-di-GMP production by *Pseudomonas aeruginosa*. J. Bacteriol. [Preprint]. 10.1128/JB.00741-18, PMID: 30642992 PMC6707927

[ref6] Baltrus D. A. Dougherty K. Diaz B. Murillo R. (2018). Evolutionary plasticity of AmrZ regulation in *Pseudomonas*. mSphere 3:e00132. 10.1128/mSphere.00132-18, PMID: 29669886 PMC5907648

[ref7] Baynham P. J. Ramsey D. M. Gvozdyev B. V. Cordonnier E. M. Wozniak D. J. (2006). The *Pseudomonas aeruginosa* ribbon-helix-helix DNA-binding protein AlgZ (AmrZ) controls twitching motility and biogenesis of type IV pili. J. Bacteriol. 188, 132–140. 10.1128/JB.188.1.132-140.2006 16352829 PMC1317580

[ref8] Baynham P. J. Wozniak D. J. (1996). Identification and characterization of AlgZ, an AlgT-dependent DNA-binding protein required for *Pseudomonas aeruginosa algD* transcription. Mol. Microbiol. 22, 97–108. 10.1111/j.1365-2958.1996.tb02659.x, PMID: 8899712

[ref9] Brencic A. McFarland K. A. McManus H. R. Castang S. Mogno I. Dove S. L.. (2009). The GacS/GacA signal transduction system of *Pseudomonas aeruginosa* acts exclusively through its control over the transcription of the RsmY and RsmZ regulatory small RNAs. Mol. Microbiol. 73, 434–445. 10.1111/j.1365-2958.2009.06782.x, PMID: 19602144 PMC2761719

[ref10] Caiazza N. C. Merritt J. H. Brothers K. M. O’Toole G. A. (2007). Inverse regulation of biofilm formation and swarming motility by *Pseudomonas aeruginosa* PA14. J. Bacteriol. 189, 3603–3612. 10.1128/JB.01685-06, PMID: 17337585 PMC1855903

[ref11] Caiazza N. C. Shanks R. M. O’Toole G. A. (2005). Rhamnolipids modulate swarming motility patterns of *Pseudomonas aeruginosa*. J. Bacteriol. 187, 7351–7361. 10.1128/JB.187.21.7351-7361.2005, PMID: 16237018 PMC1273001

[ref12] Cangelosi G. A. Palermo C. O. Laurent J. P. Hamlin A. M. Brabant W. H. (1999). Colony morphotypes on Congo red agar segregate along species and drug susceptibility lines in the *Mycobacterium avium-intracellulare* complex. Microbiology 145, 1317–1324. 10.1099/13500872-145-6-1317, PMID: 10411258

[ref13] Cheang Q. W. Xin L. Chea R. Y. F. Liang Z. X. (2019). Emerging paradigms for PilZ domain-mediated C-di-GMP signaling. Biochem. Soc. Trans. 47, 381–388. 10.1042/BST20180543, PMID: 30710060

[ref14] Chen A. I. Dolben E. F. Okegbe C. Harty C. E. Golub Y. Thao S.. (2014). *Candida albicans* ethanol stimulates *Pseudomonas aeruginosa* WspR-controlled biofilm formation as part of a cyclic relationship involving phenazines. PLoS Pathog. 10:e1004480. 10.1371/journal.ppat.1004480, PMID: 25340349 PMC4207824

[ref15] Choi K.-H. Kumar A. Schweizer H. P. (2006). A 10-min method for preparation of highly electrocompetent *Pseudomonas aeruginosa* cells: application for DNA fragment transfer between chromosomes and plasmid transformation. J. Microbiol. Methods 64, 391–397. 10.1016/j.mimet.2005.06.001, PMID: 15987659

[ref16] Choi K. H. Schweizer H. P. (2006). Mini-Tn*7* insertion in bacteria with single *att*Tn*7* sites: example *Pseudomonas aeruginosa*. Nat. Protoc. 1, 153–161. 10.1038/nprot.2006.24, PMID: 17406227

[ref17] Colvin K. M. Irie Y. Tart C. S. Urbano R. Whitney J. C. Ryder C.. (2012). The Pel and Psl polysaccharides provide *Pseudomonas aeruginosa* structural redundancy within the biofilm matrix. Environ. Microbiol. 14, 1913–1928. 10.1111/j.1462-2920.2011.02657.x, PMID: 22176658 PMC3840794

[ref18] Costaglioli P. Barthe C. Fayon M. Christoflour N. Bui S. Derlich L.. (2014). Selection of *Pseudomonas aeruginosa* reference genes for RT-qPCR analysis from sputum of cystic fibrosis patients. Mol. Cell. Probes 28, 10–12. 10.1016/j.mcp.2013.09.003, PMID: 24075879

[ref19] Deziel E. Lepine F. Milot S. Villemur R. (2003). *rhlA* is required for the production of a novel biosurfactant promoting swarming motility in *Pseudomonas aeruginosa*: 3-(3-hydroxyalkanoyloxy)alkanoic acids (HAAs), the precursors of rhamnolipids. Microbiology 149, 2005–2013. 10.1099/mic.0.26154-0, PMID: 12904540

[ref20] Frangipani E. Visaggio D. Heeb S. Kaever V. Camara M. Visca P.. (2014). The Gac/Rsm and cyclic-di-GMP signalling networks coordinately regulate iron uptake in *Pseudomonas aeruginosa*. Environ. Microbiol. 16, 676–688. 10.1111/1462-2920.12164, PMID: 23796404

[ref21] Friedman L. Kolter R. (2004). Genes involved in matrix formation in *Pseudomonas aeruginosa* PA14 biofilms. Mol. Microbiol. 51, 675–690. 10.1046/j.1365-2958.2003.03877.x, PMID: 14731271

[ref22] Ghorbal S. K. B. Chourabi K. Maalej L. Ammar A. B. Ouzari H. I. Hassen A. (2019). *Pseudomonas aeruginosa* swarmer cells adaptation toward UVc radiations. Front. Microbiol. 10:556. 10.3389/fmicb.2019.00556 31001210 PMC6454200

[ref23] Guzman L. M. Belin D. Carson M. J. Beckwith J. (1995). Tight regulation, modulation, and high-level expression by vectors containing the arabinose PBAD promoter. J. Bacteriol. 177, 4121–4130. 10.1128/jb.177.14.4121-4130.1995, PMID: 7608087 PMC177145

[ref24] Ha D. G. Kuchma S. L. O’Toole G. A. (2014). Plate-based assay for swarming motility in *Pseudomonas aeruginosa*. Methods Mol. Biol. 1149, 67–72. 10.1007/978-1-4939-0473-0_8 24818898 PMC9006052

[ref25] Ha D. G. O’Toole G. A. (2015). C-di-GMP and its effects on biofilm formation and dispersion: a *Pseudomonas aeruginosa* review. Microbiol. Spectr. 3:MB-0003-2014. 10.1128/microbiolspec.MB-0003-2014, PMID: 26104694 PMC4498269

[ref26] Hengge R. (2009). Principles of c-di-GMP signalling in bacteria. Nat. Rev. Microbiol. 7, 263–273. 10.1038/nrmicro2109, PMID: 19287449

[ref27] Hoang T. T. Karkhoff-Schweizer R. R. Kutchma A. J. Schweizer H. P. (1998). A broad-host-range Flp-FRT recombination system for site-specific excision of chromosomally-located DNA sequences: application for isolation of unmarked *Pseudomonas aeruginosa* mutants. Gene 212, 77–86. 10.1016/S0378-1119(98)00130-9, PMID: 9661666

[ref28] Jean-Pierre F. Tremblay J. Deziel E. (2016). Broth versus surface-grown cells: differential regulation of RsmY/Z small RNAs in *Pseudomonas aeruginosa* by the Gac/HptB system. Front. Microbiol. 7:2168. 10.3389/fmicb.2016.02168, PMID: 28119684 PMC5222819

[ref29] Jones C. J. Newsom D. Kelly B. Irie Y. Jennings L. K. Xu B.. (2014). ChIP-Seq and RNA-Seq reveal an AmrZ-mediated mechanism for cyclic di-GMP synthesis and biofilm development by *Pseudomonas aeruginosa*. PLoS Pathog. 10:e1003984. 10.1371/journal.ppat.1003984, PMID: 24603766 PMC3946381

[ref30] Jones C. J. Ryder C. R. Mann E. E. Wozniak D. J. (2013). AmrZ modulates *Pseudomonas aeruginosa* biofilm architecture by directly repressing transcription of the *psl* operon. J. Bacteriol. 195, 1637–1644. 10.1128/JB.02190-12, PMID: 23354748 PMC3624555

[ref31] Kearns D. (2010). A field guide to bacterial swarming motility. Nat. Rev. Microbiol. 8, 634–644. 10.1038/nrmicro2405, PMID: 20694026 PMC3135019

[ref32] Kirisits M. J. Prost L. Starkey M. Parsek M. R. (2005). Characterization of colony morphology variants isolated from *Pseudomonas aeruginosa* biofilms. Appl. Environ. Microbiol. 71, 4809–4821. 10.1128/AEM.71.8.4809-4821.2005, PMID: 16085879 PMC1183349

[ref33] Ko J. Ryu K. S. Kim H. Shin J. S. Lee J. O. Cheong C.. (2010). Structure of PP4397 reveals the molecular basis for different c-di-GMP binding modes by PilZ domain proteins. J. Mol. Biol. 398, 97–110. 10.1016/j.jmb.2010.03.007, PMID: 20226196

[ref34] Kohler T. Curty L. K. Barja F. van Delden C. Pechere J. C. (2000). Swarming of *Pseudomonas aeruginosa* is dependent on cell-to-cell signaling and requires flagella and pili. J. Bacteriol. 182, 5990–5996. 10.1128/JB.182.21.5990-5996.2000, PMID: 11029417 PMC94731

[ref35] Kong W. Chen L. Zhao J. Shen T. Surette M. G. Shen L.. (2013). Hybrid sensor kinase PA1611 in *Pseudomonas aeruginosa* regulates transitions between acute and chronic infection through direct interaction with RetS. Mol. Microbiol. 88, 784–797. 10.1111/mmi.12223, PMID: 23560772

[ref36] Krasteva P. V. Giglio K. M. Sondermann H. (2012). Sensing the messenger: the diverse ways that bacteria signal through c-di-GMP. Protein Sci. 21, 929–948. 10.1002/pro.2093, PMID: 22593024 PMC3403432

[ref37] Kuchma S. L. Brothers K. M. Merritt J. H. Liberati N. T. Ausubel F. M. O’Toole G. A. (2007). BifA, a cyclic-Di-GMP phosphodiesterase, inversely regulates biofilm formation and swarming motility by *Pseudomonas aeruginosa* PA14. J. Bacteriol. 189, 8165–8178. 10.1128/JB.00586-07, PMID: 17586641 PMC2168662

[ref38] Kuchma S. L. Delalez N. J. Filkins L. M. Snavely E. A. Armitage J. P. O’Toole G. A. (2015). Cyclic Di-GMP-mediated repression of swarming motility by *Pseudomonas aeruginosa* PA14 requires the MotAB stator. J. Bacteriol. 197, 420–430. 10.1128/JB.02130-14, PMID: 25349157 PMC4285984

[ref39] Kuchma S. L. Griffin E. F. O’Toole G. A. (2012). Minor pilins of the type IV pilus system participate in the negative regulation of swarming motility. J. Bacteriol. 194, 5388–5403. 10.1128/JB.00899-12, PMID: 22865844 PMC3457191

[ref40] Lee V. T. Matewish J. M. Kessler J. L. Hyodo M. Hayakawa Y. Lory S. (2007). A cyclic-di-GMP receptor required for bacterial exopolysaccharide production. Mol. Microbiol. 65, 1474–1484. 10.1111/j.1365-2958.2007.05879.x, PMID: 17824927 PMC2170427

[ref41] Lewis K. A. Baker A. E. Chen A. I. Harty C. E. Kuchma S. L. O’Toole G. A.. (2019). Ethanol decreases *Pseudomonas aeruginosa* flagellar motility through the regulation of flagellar stators. J. Bacteriol. [Preprint]. 10.1128/JB.00285-19, PMID: 31109994 PMC6707923

[ref42] Li K. Xu C. Jin Y. Sun Z. Liu C. Shi J.. (2013). SuhB is a regulator of multiple virulence genes and essential for pathogenesis of *Pseudomonas aeruginosa*. MBio 4, e00419–e00413. 10.1128/mBio.00419-13, PMID: 24169572 PMC3809559

[ref43] Li K. Yang G. Debru A. B. Li P. Zong L. Li P.. (2017). SuhB regulates the motile-sessile switch in *Pseudomonas aeruginosa* through the Gac/Rsm pathway and c-di-GMP signaling. Front. Microbiol. 8:1045. 10.3389/fmicb.2017.01045, PMID: 28642753 PMC5462983

[ref44] Livak K. J. Schmittgen T. D. (2001). Analysis of relative gene expression data using real-time quantitative PCR and the 2^−ΔΔCT^ method. Methods 25, 402–408. 10.1006/meth.2001.1262, PMID: 11846609

[ref45] Ma L. Wang J. Wang S. Anderson E. M. Lam J. S. Parsek M. R.. (2012). Synthesis of multiple *Pseudomonas aeruginosa* biofilm matrix exopolysaccharides is post-transcriptionally regulated. Environ. Microbiol. 14, 1995–2005. 10.1111/j.1462-2920.2012.02753.x, PMID: 22513190 PMC4446059

[ref46] Martinez-Granero F. Redondo-Nieto M. Vesga P. Martin M. Rivilla R. (2014). AmrZ is a global transcriptional regulator implicated in iron uptake and environmental adaption in *P. fluorescens* F113. BMC Genomics 15:237. 10.1186/1471-2164-15-237, PMID: 24670089 PMC3986905

[ref47] McCarter L. L. Gomelsky M. (2015). Fifty ways to inhibit motility via cyclic Di-GMP: the emerging *Pseudomonas aeruginosa* swarming story. J. Bacteriol. 197, 406–409. 10.1128/JB.02483-14, PMID: 25448814 PMC4285990

[ref48] Merighi M. Lee V. T. Hyodo M. Hayakawa Y. Lory S. (2007). The second messenger bis-(3’-5’)-cyclic-GMP and its PilZ domain-containing receptor Alg44 are required for alginate biosynthesis in *Pseudomonas aeruginosa*. Mol. Microbiol. 65, 876–895. 10.1111/j.1365-2958.2007.05817.x, PMID: 17645452

[ref49] Merritt J. H. Ha D. G. Cowles K. N. Lu W. Morales D. K. Rabinowitz J. (2010). Specific control of *Pseudomonas aeruginosa* surface-associated behaviors by two c-di-GMP diguanylate cyclases. MBio 1, e00183–e00110. 10.1128/mBio.00183-10 20978535 PMC2957078

[ref50] Moscoso J. A. Mikkelsen H. Heeb S. Williams P. Filloux A. (2011). The *Pseudomonas aeruginosa* sensor RetS switches type III and type VI secretion via c-di-GMP signalling. Environ. Microbiol. 13, 3128–3138. 10.1111/j.1462-2920.2011.02595.x, PMID: 21955777

[ref51] Murray T. S. Kazmierczak B. I. (2008). *Pseudomonas aeruginosa* exhibits sliding motility in the absence of type IV pili and flagella. J. Bacteriol. 190, 2700–2708. 10.1128/JB.01620-07, PMID: 18065549 PMC2293233

[ref52] Murray T. S. Ledizet M. Kazmierczak B. I. (2010). Swarming motility, secretion of type 3 effectors and biofilm formation phenotypes exhibited within a large cohort of *Pseudomonas aeruginosa* clinical isolates. J. Med. Microbiol. 59, 511–520. 10.1099/jmm.0.017715-0, PMID: 20093376 PMC2855384

[ref53] Newell P. D. Yoshioka S. Hvorecny K. L. Monds R. D. O’Toole G. A. (2011). Systematic analysis of diguanylate cyclases that promote biofilm formation by *Pseudomonas fluorescens* Pf0-1. J. Bacteriol. 193, 4685–4698. 10.1128/JB.05483-11, PMID: 21764921 PMC3165641

[ref54] Orr M. W. Lee V. T. (2016). A PilZ domain protein for chemotaxis adds another layer to c-di-GMP-mediated regulation of flagellar motility. Sci. Signal. 9:fs16. 10.1126/scisignal.aai8859, PMID: 27811181

[ref55] Overhage J. Bains M. Brazas M. D. Hancock R. E. (2008). Swarming of *Pseudomonas aeruginosa* is a complex adaptation leading to increased production of virulence factors and antibiotic resistance. J. Bacteriol. 190, 2671–2679. 10.1128/JB.01659-07, PMID: 18245294 PMC2293252

[ref56] Partridge J. D. Harshey R. M. (2013). Swarming: flexible roaming plans. J. Bacteriol. 195, 909–918. 10.1128/JB.02063-12, PMID: 23264580 PMC3571328

[ref57] Paul K. Nieto V. Carlquist W. C. Blair D. F. Harshey R. M. (2010). The c-di-GMP binding protein YcgR controls flagellar motor direction and speed to affect chemotaxis by a “backstop brake” mechanism. Mol. Cell 38, 128–139. 10.1016/j.molcel.2010.03.001, PMID: 20346719 PMC2929022

[ref58] Petrova O. E. Cherny K. E. Sauer K. (2014). The *Pseudomonas aeruginosa* diguanylate cyclase GcbA, a homolog of *P. fluorescens* GcbA, promotes initial attachment to surfaces, but not biofilm formation, via regulation of motility. J. Bacteriol. 196, 2827–2841. 10.1128/JB.01628-14, PMID: 24891445 PMC4135668

[ref59] Prada-Ramirez H. A. Perez-Mendoza D. Felipe A. Martinez-Granero F. Rivilla R. Sanjuan J.. (2016). AmrZ regulates cellulose production in *Pseudomonas syringae* pv. Tomato DC3000. Mol. Microbiol. 99, 960–977. 10.1111/mmi.13278, PMID: 26564578

[ref60] Price K. E. Naimie A. A. Griffin E. F. Bay C. O’Toole G. A. (2016). Tobramycin-treated *Pseudomonas aeruginosa* PA14 enhances *Streptococcus constellatus* 7155 biofilm formation in a cystic fibrosis model system. J. Bacteriol. 198, 237–247. 10.1128/JB.00705-15 26483523 PMC4751783

[ref61] Pryor E. E. Jr. Waligora E. A. Xu B. Dellos-Nolan S. Wozniak D. J. Hollis T. (2012). The transcription factor AmrZ utilizes multiple DNA binding modes to recognize activator and repressor sequences of *Pseudomonas aeruginosa* virulence genes. PLoS Pathog. 8:e1002648. 10.1371/journal.ppat.1002648, PMID: 22511872 PMC3325190

[ref62] Rashid M. H. Kornberg A. (2000). Inorganic polyphosphate is needed for swimming, swarming, and twitching motilities of *Pseudomonas aeruginosa*. Proc. Natl. Acad. Sci. USA 97, 4885–4890. 10.1073/pnas.060030097 10758151 PMC18327

[ref63] Romling U. Galperin M. Y. Gomelsky M. (2013). Cyclic di-GMP: the first 25 years of a universal bacterial second messenger. Microbiol. Mol. Biol. Rev. 77, 1–52. 10.1128/MMBR.00043-12, PMID: 23471616 PMC3591986

[ref64] Schirmer T. Jenal U. (2009). Structural and mechanistic determinants of c-di-GMP signalling. Nat. Rev. Microbiol. 7, 724–735. 10.1038/nrmicro2203, PMID: 19756011

[ref65] Siegmund I. Wagner F. (1991). New method for detecting rhamnolipids excreted by *Pseudomonas* species during growth on mineral agar. Biotechnol. Tech. 5, 265–268. 10.1007/BF02438660

[ref66] Silby M. W. Winstanley C. Godfrey S. A. Levy S. B. Jackson R. W. (2011). Pseudomonas genomes: diverse and adaptable. FEMS Microbiol. Rev. 35, 652–680. 10.1111/j.1574-6976.2011.00269.x, PMID: 21361996

[ref67] Spangler C. Bohm A. Jenal U. Seifert R. Kaever V. (2010). A liquid chromatography-coupled tandem mass spectrometry method for quantitation of cyclic di-guanosine monophosphate. J. Microbiol. Methods 81, 226–231. 10.1016/j.mimet.2010.03.020, PMID: 20385176

[ref68] Tart A. H. Blanks M. J. Wozniak D. J. (2006). The AlgT-dependent transcriptional regulator AmrZ (AlgZ) inhibits flagellum biosynthesis in mucoid, nonmotile *Pseudomonas aeruginosa* cystic fibrosis isolates. J. Bacteriol. 188, 6483–6489. 10.1128/JB.00636-06, PMID: 16952938 PMC1595476

[ref69] Toutain C. M. Zegans M. E. O’Toole G. A. (2005). Evidence for two flagellar stators and their role in the motility of *Pseudomonas aeruginosa*. J. Bacteriol. 187, 771–777. 10.1128/JB.187.2.771-777.2005, PMID: 15629949 PMC543560

[ref70] Tremblay J. Deziel E. (2010). Gene expression in *Pseudomonas aeruginosa* swarming motility. BMC Genomics 11:587. 10.1186/1471-2164-11-587, PMID: 20961425 PMC3091734

[ref71] Valentini M. Filloux A. (2016). Biofilms and cyclic di-GMP (c-di-GMP) Signaling: lessons from *Pseudomonas aeruginosa* and other bacteria. J. Biol. Chem. 291, 12547–12555. 10.1074/jbc.R115.711507, PMID: 27129226 PMC4933438

[ref72] Visaggio D. Pasqua M. Bonchi C. Kaever V. Visca P. Imperi F. (2015). Cell aggregation promotes pyoverdine-dependent iron uptake and virulence in *Pseudomonas aeruginosa*. Front. Microbiol. 6:902. 10.3389/fmicb.2015.00902, PMID: 26379660 PMC4552172

[ref73] West S. Schweizer H. Dall C. Sample A. Runyen-Janecky L. (1994). Construction of improved *Escherichia*-*Pseudomonas* shuttle vectors derived from pUC18/19 and sequence of the region required for their replication in *Pseudomonas aeruginosa*. Gene 148, 81–86. 10.1016/0378-1119(94)90237-2, PMID: 7926843

[ref74] Wolfe A. J. Visick K. L. (2008). Get the message out: cyclic-Di-GMP regulates multiple levels of flagellum-based motility. J. Bacteriol. 190, 463–475. 10.1128/JB.01418-07, PMID: 17993515 PMC2223684

[ref75] Xu L. Venkataramani P. Ding Y. Liu Y. Deng Y. Yong G. L. (2016a). A cyclic di-GMP-binding adaptor protein interacts with histidine kinase to regulate two-component Signaling. J. Biol. Chem. 291, 16112–16123. 10.1074/jbc.M116.730887 27231351 PMC4965561

[ref76] Xu L. Xin L. Zeng Y. Yam J. K. Ding Y. Venkataramani P. (2016b). A cyclic di-GMP-binding adaptor protein interacts with a chemotaxis methyltransferase to control flagellar motor switching. Sci. Signal. 9:ra102. 10.1126/scisignal.aaf7584 27811183

[ref77] Yan J. Deforet M. Boyle K. E. Rahman R. Liang R. Okegbe C.. (2017). Bow-tie signaling in c-di-GMP: machine learning in a simple biochemical network. PLoS Comput. Biol. 13:e1005677. 10.1371/journal.pcbi.1005677, PMID: 28767643 PMC5555705

[ref78] Yeung A. T. Parayno A. Hancock R. E. (2012). Mucin promotes rapid surface motility in *Pseudomonas aeruginosa*. MBio 3, e00073–e00012. 10.1128/mBio.00073-12 22550036 PMC3569861

[ref79] Yeung A. T. Torfs E. C. Jamshidi F. Bains M. Wiegand I. Hancock R. E.. (2009). Swarming of *Pseudomonas aeruginosa* is controlled by a broad spectrum of transcriptional regulators, including MetR. J. Bacteriol. 191, 5592–5602. 10.1128/JB.00157-09, PMID: 19592586 PMC2737960

[ref80] Zorraquino V. Garcia B. Latasa C. Echeverz M. Toledo-Arana A. Valle J.. (2013). Coordinated cyclic-di-GMP repression of *Salmonella* motility through YcgR and cellulose. J. Bacteriol. 195, 417–428. 10.1128/JB.01789-12, PMID: 23161026 PMC3554008

