# Supplementary material for: AmrZ Regulates Swarming Motility Through Cyclic di-GMP-Dependent Motility Inhibition and Controlling Pel Polysaccharide Production in Pseudomonas aeruginosa PA14
Source: Front Microbiol. 2019 Aug 14;10:1847. doi: 10.3389/fmicb.2019.01847 (PMC6707383; doi:10.3389/fmicb.2019.01847)
Supplement: Supplementary file 1 [file Data_Sheet_1.docx]

# Supplementary Figures and Tables

## Supplementary Figures


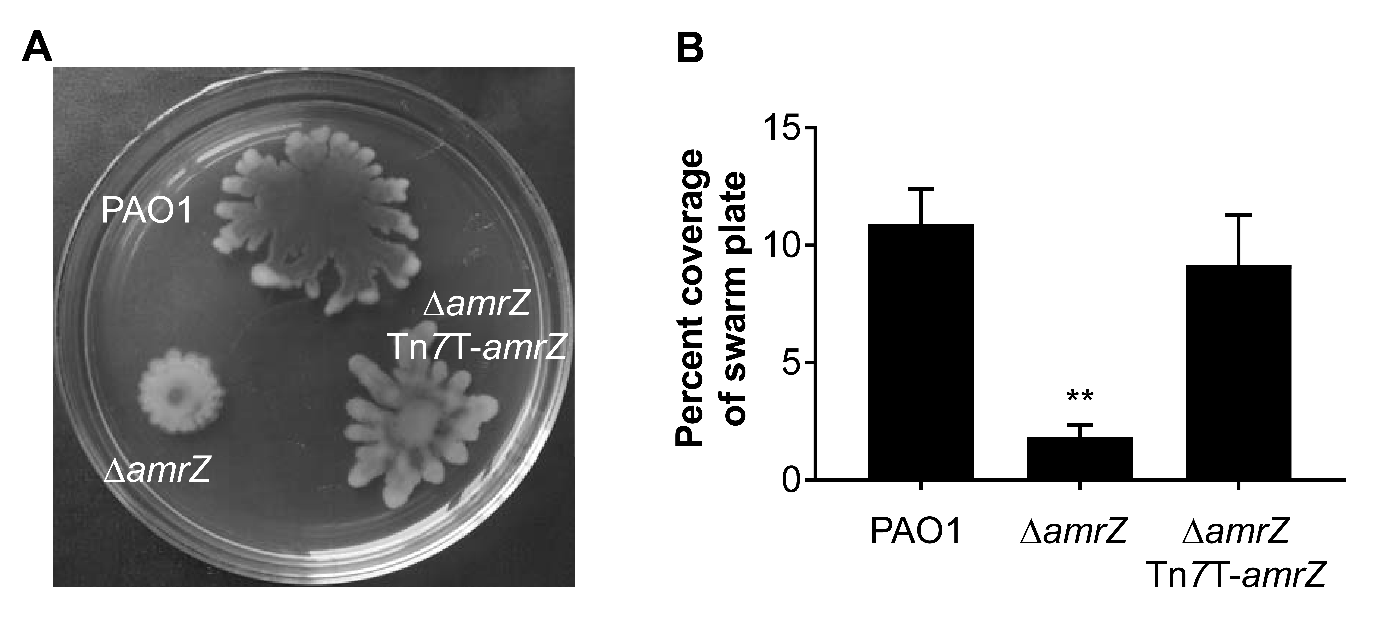


**Figure S1.** AmrZ is required for the swarming motility of *P. aeruginosa* PAO1. **(A)** Swarming motility of the WT PAO1 strain, the Δ*amrZ* mutant and the single-copy complemented strain of the Δ*amrZ* mutant carrying an *amrZ* gene inserted into chromosome by mini-Tn*7*T. **(B)** Quantification of percent coverage of swarm plates for the indicated strains. Assays were repeated at least in triplicate. **, significantly different from PAO1 (*P* < 0.01).


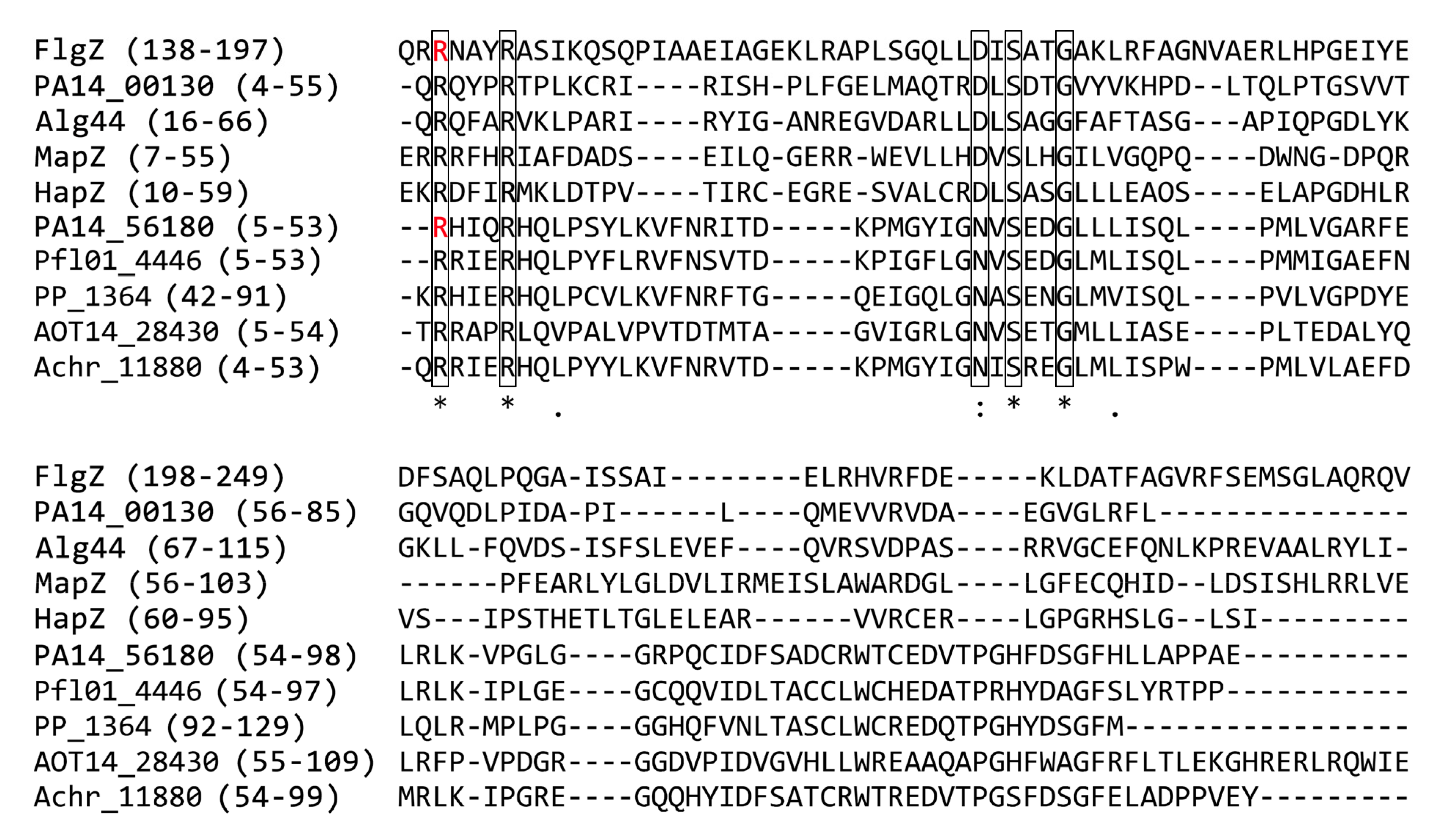


**Figure S2.** Multiple sequence alignment of PilZ domains of *P. aeruginosa* FlgZ, PA14_00130, Alg44, MapZ, HapZ, PA14_56180 and its orthologs from *Pseudomonas fluorescens* (Pfl01_4446, 59.7% identity to PA14_56180), *Pseudomonas putida* (PP_1364, 58.5% identity), *Stenotrophomonas acidaminiphila* (AOT14_28430, 39.6% identity) and *Azotobacter chroococcum* (71.4% identity). The alignment was generated by Clustal Omega (https://www.ebi.ac.uk/Tools/ msa/clustalo/) using the complete PilZ domain (indicated by amino acid sequence number in parentheses) of each protein assigned by a Pfam domain search (http://pfam.xfam.org/search). The conservation of residues was determined by Clustal Omega and the highly conserved residues are highlighted in box. * and : represent a fully conserved residue or a residue with strongly similar properties, respectively. The arginine (R) residue in the RXXXR motif of FlgZ and PA14_56180 that was substituted by alanine (A) for site-directed mutagenesis are highlighted in red.


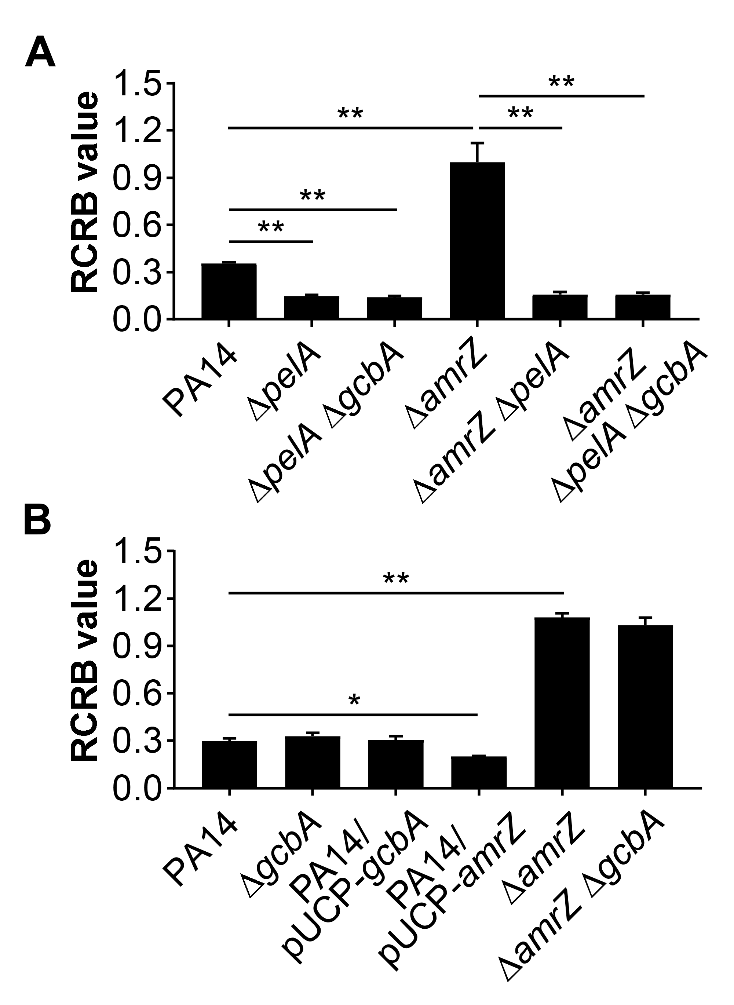


**Figure S3.** Quantification of Congo red binding ability of the indicated strains in **(A)** and **(B)**. Colonies scraped from CR plates were suspended in deionized water and the OD_660_ was measured. Cells were then pelleted, resuspended in acetone, and the CR dye was extracted at room temperature for 2 h. The relative Congo red binding (RCRB) value was calculated by dividing the OD_488_ of the acetone extracts by the OD_660_ of the cell suspension before acetone extraction. Significance was determined using one-way ANOVA followed by Tukey's multiple comparison test. * *P* < 0.05, ** *P* < 0.01.

## Supplementary Tables

**Table S1.** Bacterial strains and plasmids used in this study.

| **Strains/Plasmids** | **Genotype or phenotype** | **Reference**  **or origin** |
| --- | --- | --- |
| **Strains** |  |  |
| ***P. aeruginosa*** **strains** |  |  |
| PA14 | UCBPP-PA14 wild-type strain | (Liberati et al., 2006) |
| PA14 ∆*amrZ* | *amrZ* deletion mutant derived from strain PA14 | This study |
| PA14 ∆*rhlA* | *rhlA* deletion mutant derived from strain PA14 | Lab collection |
| PA14 ∆*gcbA* | *gcbA* deletion mutant derived from strain PA14 | This study |
| PA14 ∆*amrZ* ∆*gcbA* | PA14 with deletions of *amrZ* and *gcbA* | This study |
| PA14 ∆*PA14_00130* | *PA14_00130* deletion mutant | This study |
| PA14 ∆*hapZ* | *hapZ* deletion mutant | This study |
| PA14 ∆*pilZ* | *pilZ* deletion mutant | This study |
| PA14 ∆*PA14_25420* | *PA14_25420* deletion mutant | This study |
| PA14 ∆*flgZ* | *flgZ* deletion mutant | This study |
| PA14 ∆*alg44* | *alg44* deletion mutant | This study |
| PA14 ∆*PA14_56180* | *PA14_56180* deletion mutant | This study |
| PA14 ∆*mapZ* | *mapZ* deletion mutant | This study |
| PA14 ∆*flgZ* ∆*PA14_56180* | PA14 with deletions of *flgZ* and *PA14_56180* | This study |
| PA14 ∆*amrZ* ∆*flgZ* | PA14 with deletions of *amrZ* and *flgZ* | This study |
| PA14 ∆*amrZ* ∆*PA14_56180* | PA14 with deletions of *amrZ* and *PA14_56180* | This study |
| PA14 ∆*amrZ* ∆*flgZ* ∆*PA14_56180* | PA14 with deletions of *amrZ*, *flgZ* and *PA14_56180* | This study |

**Table S1,** continued

| **Strains/Plasmids** | **Genotype or phenotype** | **Reference**  **or origin** |
| --- | --- | --- |
| PA14 ∆*pelA* | *pelA* deletion mutant | This study |
| PA14 ∆*pelA* ∆*gcbA* | PA14 with deletions of *pelA* and *gcbA* | This study |
| PA14 ∆*amrZ* ∆*pelA* | PA14 with deletions of *amrZ* and *pelA* | This study |
| PA14 ∆*amrZ* ∆*pelA* ∆*gcbA* | PA14 with deletions of *amrZ*, *pelA* and *gcbA* | This study |
| PAO1 | Wild-type *P. aeruginosa* PAO1 strain | (Stover et al., 2000) |
| PAO1 ∆*amrZ* | PAO1 with a *amrZ* deletion | This study |
|  |  |  |
| ***E. coli* strains** |  |  |
| DH5α | φ80d*lacZ*∆M15 ∆(*lacZYA-argF*)*U169 recA1 endA1 hsdR17*(r_K_^-^ m _K_^-^) *supE44 thi-1 gyrA relA1* | Lab collection |
| S17-1 | *thi pro hdsR hdsM* ^+^ *recA*; chromosomal insertion of RP4-2 (Tc::Mu Km::Tn*7*) | Lab collection |
|  |  |  |
| **Plasmids** |  |  |
| pEX18Tc | Gene replacement vector; Tc^r^, *oriT*^+^, *sacB*^+^ | (Hoang et al., 1998) |
| pUC18T-mini-Tn*7*T-Gm | mini-Tn*7* base vector for insertion into chromosome *att*Tn*7* site; Gm^r^ | (Choi and Schweizer, 2006) |
| pTNS2 | Helper plasmid, for gene insertion in chromosome; Amp^r^ | (Choi and Schweizer, 2006) |
| pUCP20 | Shuttle vector between *E. coli* and *P. aeruginosa;* Amp^r^ | (West et al., 1994) |
| pTn*7*T-*amrZ* | *amrZ* gene of PA14 driven by its own promoter on pUC18T-mini-Tn*7*T-Gm; Gm^r^ | This study |

**Table S1,** continued

| **Strains/Plasmids** | **Genotype or phenotype** | **Reference**  **or origin** |
| --- | --- | --- |
| pTn*7*T-*amrZ-*PAO1 | *amrZ* gene of PAO1 driven by its own promoter on pUC18T-mini-Tn*7*T-Gm; Gm^r^ | This study |
| pUCP*-amrZ* | AmrZ expression plasmid on pUCP20 driven by *lac* promoter; Amp^r^ | This study |
| pUCP-*Zsgreen1* | Zsgreen1 expression plasmid on pUCP20 driven by *lac* promoter; Amp^r^, GFP^+^ | (Li et al., 2017) |
| pUCP-*2133* | PA2133 expression plasmid on pUCP20 driven by *lac* promoter; Amp^r^ | This study |
| pUCP*-gcbA* | GcbA expression plasmid on pUCP20 driven by *lac* promoter; Amp^r^ | This study |
| pUC18T-mini-Tn*7*T-Gm-BAD | *araC*-P*_BAD_* cloned in pUC18T-mini-Tn*7*T-Gm for construction of strains with chromosomal P*_BAD_* expression cassette; Gm^r^ | This study |
| pTn*7*T-*flgZ* (WT)-FLAG | Plasmid harboring FLAG-tagged WT FlgZ under the control of the P*_BAD_* promoter cloned in pUC18T-mini-Tn*7*T-Gm-BAD; Gm^r^ | This study |
| pTn*7*T-*flgZ* (R140A)-FLAG | Plasmid harboring FLAG-tagged FlgZ (R140A) under the control of the P*_BAD_* promoter cloned in pUC18T-mini- Tn*7*T-Gm-BAD; Gm^r^ | This study |
| pTn*7*T-*PA14_56180* (WT)-FLAG | Plasmid harboring FLAG-tagged WT PA14_56180 under the control of the P*_BAD_* promoter cloned in pUC18T-mini- Tn*7*T-Gm-BAD; Gm^r^ | This study |
| pTn*7*T- *PA14_56180* (R5A)-FLAG | Plasmid harboring FLAG-tagged PA14_56180 (R5A) under the control of the P*_BAD_* promoter cloned in pUC18T-mini- Tn*7*T-Gm-BAD; Gm^r^ | This study |

**Table S2.** Primers used in this study.

| **Primers** | **Sequence (5' to 3')** | **Use** |
| --- | --- | --- |
| pEX-*amrZ*-up-F | CCACCGTCTAGAGGCAATAGCGCAACGCGTAG | Constructing *amrZ* mutant |
| pEX-*amrZ*-up-R | GTCAGCGGTACCGCTGGAGTAGGTAGGAGTTG |  |
|  |  |  |
| pEX-*amrZ*-down-F | AGCAGAGGTACCTGGTCGCCCTGATCGCACAC | Constructing *amrZ* mutant |
| pEX-*amrZ*-down-R | CGCAGGAAGCTTCGTCACGTGCGCAGATGAAG |  |
|  |  |  |
| pTn*7*T-*amrZ*-F | CGTTCTGGATCCAATCGGTTGCACGAAGAC | Constructing pTn*7*T-*amrZ* plasmid |
| pTn*7*T-*amrZ*-R | GTGGCGAAGCTTGGATAACGCCACCGCTACAG |  |
|  |  |  |
| pUCP-*amrZ*-F | TCAGGTACCCGAGAACAATGAACGCTTCCTC | Constructing pUCP*-amrZ* plasmid |
| pUCP-*amrZ*-R | ACGAAGCTTCTTCGGCGCTCAGGCCTG |  |
|  |  |  |
| pUCP-*2133*-F | GGCGTCGGATCCTCACACAGGAAACTACAGTGAACGGTTCCCCAC | Constructing pUCP-*2133* plasmid |
| pUCP-*2133*-R | GCTCCCAAGCTTGGAAGGCTGATTGCTCTGTT |  |
|  |  |  |
| pEX-*gcbA*-up-F | TGGACTGGTACCGCGAACGGGCGAAATCCTTG | Constructing *gcbA* mutant |
| pEX-*gcbA*-up-R | GGTCCAGGATCCGGTCATCGTGCTCGGTCATC |  |
|  |  |  |
| pEX-*gcbA*-down-F | TCGAGGATCCTCGTCCCGACGCTCGATAATGG | Constructing *gcbA* mutant |
| pEX-*gcbA*-down-R | ATCCCAAGCTTGATCAGGCGTACCTGGCCTTG |  |
|  |  |  |
| pUCP-*gcbA*-F | GCTGGCGGTACCGGCGATAGGTGATAGCATAG | Constructing pUCP-*gcbA* plasmid |
| pUCP-*gcbA*-R | GAGCGGAAGCTTCGCAGGTTATTTCCGTTTGG |  |
|  |  |  |
| pEX-*PA14_00130*-up-F | CCCAAGCTTCTGGCTGATGTGGAAATTGC | Constructing *PA14_00130* mutant |

**Table S2,** continued

| **Primers** | **Sequence (5' to 3')** | **Use** |
| --- | --- | --- |
| pEX-*PA14_00130*-up-R | CGGGGTACCTACGGATTCGGCACTTGAGC | Constructing *PA14_00130* mutant |
|  |  |  |
| pEX-*PA14_00130*-down-F | CGGGGTACCGCGAAGCCTGAACGGTGATG | Constructing *PA14_00130* mutant |
| pEX-*PA14_00130*-down-R | CTAGTCTAGACTGCCTCGATGAGCCTCAAG |  |
|  |  |  |
| pEX-*hapZ*-up-F | CCCAAGCTTCGTTGCGCCTCGACTCATAG | Constructing *hapZ* mutant |
| pEX-*hapZ*-up-R | CGGGGTACCATGGGCTGATCCTCATGGTC |  |
|  |  |  |
| pEX-*hapZ*-down-F | CGGGGTACCTCCACTGAAACGACGAAAGG | Constructing *hapZ* mutant |
| pEX-*hapZ*-down-R | CTAGTCTAGATGCCCGTAGCTGTCCATGTC |  |
|  |  |  |
| pEX-*pilZ*-up-F | CTAGTCTAGACCCACGCCTATCTGCTCTAC | Constructing *pilZ* mutant |
| pEX-*pilZ*-up-R | CGCGGATCCCCAGATTGGGTGGCAAACTC |  |
|  |  |  |
| pEX-*pilZ*-down-F | CGCGGATCCCACGATGTAACGCTAGACAG | Constructing *pilZ* mutant |
| pEX-*pilZ*-down-R | CCCAAGCTTCCCACGAACATGACCAATAC |  |
|  |  |  |
| pEX-*PA14_25420*-up-F | CTAGTCTAGAAAATCCAGGCGCTGACGTTG | Constructing *PA14_25420* mutant |
| pEX-*PA14_25420*-up-R | CGCGGATCCGCAGCCATTTCGTCTCCTTC |  |
|  |  |  |
| pEX-*PA14_25420*-down-F | CGCGGATCCTCTTCGACCTGAGATGTACC | Constructing *PA14_25420* mutant |

**Table S2,** continued

| **Primers** | **Sequence (5' to 3')** | **Use** |
| --- | --- | --- |
| pEX-*PA14_25420*-down-R | CCCAAGCTTATCAGCGCCGGTTCGTTGAC | Constructing *PA14_25420* mutant |
|  |  |  |
| pEX-*flgZ*-up-F | CTAGTCTAGACGGCGGGTTTCTCTAAACTG | Constructing *flgZ* mutant |
| pEX-*flgZ*-up-R | CGCGGATCCCGGGTTGGGCACCTTCAATG |  |
|  |  |  |
| pEX-*flgZ*-down-F | CGCGGATCCATCGCTTCGTCTACCAGTTG | Constructing *flgZ* mutant |
| pEX-*flgZ*-down-R | CCCAAGCTTGGCGCTAACATGCTATATGC |  |
|  |  |  |
| pEX-*alg44*-up-F | CTAGTCTAGACACGGCGTGGTGAAGAAGAC | Constructing *alg44* mutant |
| pEX-*alg44*-up-R | CGCGGATCCTGACGTTGACGGCTGTATTC |  |
|  |  |  |
| pEX-*alg44*-down-F | CGCGGATCCCTGCTGAACAAGGCCGTGAC | Constructing *alg44* mutant |
| pEX-*alg44*-down-R | CCCAAGCTTCCCACTTGCCGTCGTAGTAG |  |
|  |  |  |
| pEX-*PA14_56180*-up-F | CTAGTCTAGACGCCGATACAGTTCCCAACG | Constructing *PA14_56180* mutant |
| pEX-*PA14_56180*-up-R | CGCGGATCCCGCTGGATATGTCGTTGAAG |  |
|  |  |  |
| pEX-*PA14_56180*-down-F | CGCGGATCCTCGGTCTGATCCGTCAAAGC | Constructing *PA14_56180* mutant |
| pEX-*PA14_56180*-down-R | CCCAAGCTTAAAGCACCGAGGTCGGCTAC |  |
|  |  |  |
| pEX-*mapZ*-up-F | CTAGTCTAGAATGGCGGTAGGCGCAATGTC | Constructing *mapZ* mutant |

**Table S2,** continued

| **Primers** | **Sequence (5' to 3')** | **Use** |
| --- | --- | --- |
| pEX-*mapZ*-up-R | CGCGGATCCCACTCATGTCGCGATCCCTC | Constructing *mapZ* mutant |
|  |  |  |
| pEX-*mapZ*-down-F | CGCGGATCCCGACTGAGGCGGGTCACTCG | Constructing *mapZ* mutant |
| pEX-*mapZ*-down-R | CCCAAGCTTTGTCGTTGCCGGAAGACAAG |  |
|  |  |  |
| pEX-*pelA*-up-F | CTAGTCTAGATGGTACCTGCAACAGATCAC | Constructing *pelA* mutant |
| pEX-*pelA*-up-R | CCGGAATTCGCACGGCGATTCCTTTCTTG |  |
|  |  |  |
| pEX-*pelA*-down-F | CCGGAATTCCGTTCGAGTTGCCGATGGAG | Constructing *pelA* mutant |
| pEX-*pelA*-down-R | CCCAAGCTTCAGGCCAGGGTTGACTCATC |  |
|  |  |  |
| pTn*7*T-*flgZ* WT-F | CCCAAGCTTACCCGACCAGCACACGTATC | Constructing pTn*7*T-*flgZ* (WT)-FLAG plasmid |
| pTn*7*T-*flgZ* WT-FLAG-R | CGGGGTACCTCACTTGTCGTCATCGTCCTTGTAGTCTCCGAACAGTTCGTCTTTCTC |  |
|  |  |  |
| pTn*7*T-*flgZ* R140A- UP-R | GTAGGCGTTGGCGCGCTGGTG | Constructing pTn*7*T-*flgZ* (R140A)-FLAG plasmid |
| pTn*7*T-*flgZ* R140A- DN-F | CACCAGCGCGCCAACGCCTAC |  |
|  |  |  |
| pTn*7*T-*PA14_56180* WT-F | CCCAAGCTTCGCTTGCGGCAACAGTTGTC | Constructing pTn*7*T-*PA14_56180* (WT)-FLAG plasmid |
| pTn*7*T-*PA14_56180* WT- FLAG-R | CGGGGTACCTCACTTGTCGTCATCGTCCTTGTAGTCTCCGACCGACTGCGGCTGGCG |  |

**Table S2,** continued

| **Primers** | **Sequence (5' to 3')** | **Use** |
| --- | --- | --- |
| pTn*7*T-*PA14_56180* R5A- UP-R | CTGGATATGTGCTTGAAGACC | Constructing pTn*7*T- *PA14_56180* (R5A)-FLAG plasmid |
| pTn*7*T-*PA14_56180* R5A- DN-F | GGTCTTCAAGCACATATCCAG |  |
|  |  |  |
| *PA14_26910*-F | AGTTTCCAGCGCATCCAGTT | Real time PCR |
| *PA14_26910*-R | CGGGATGGAAGACGAATTG |  |
|  |  |  |
| *PA14_20860*-F | GCTTGCAGTTCCTCAACGAG | Real time PCR |
| *PA14_20860*-R | CACCAGGAAATTCAGGTAGGG |  |
|  |  |  |
| *amrZ*-F | AGCATGAACTCCGAGATC | Real time PCR |
| *amrZ*-R | AACACCGAGATTGTCTTG |  |
|  |  |  |
| *gcbA* -F | TCGATATCAAGAGCATGG | Real time PCR |
| *gcbA* -R | GACGATATACCTCGATCC |  |
| *flgZ*-F | ACAACTGCTGGATATCTC | Real time PCR |
| *flgZ*-R | GAAGTCTTCGTAGATCTC |  |
|  |  |  |
| *PA14_56180*-F | GGTCTTCAACGACATATCCAG | Real time PCR |
| *PA14_56180*-R | ATCGGCTTGTCGGTGATG |  |
|  |  |  |
| *pelA*-F | TTCAGCCATCCGTTCTTC | Real time PCR |
| *pelA*-R | CCATCTTGTAGCCATACTG |  |
|  |  |  |
| *pelG*-F | CAACTTCGTGGTGCTCTG | Real time PCR |
| *pelG*-R | CAGGATGCGTTTGTAGGC |  |

# References

Choi, K.H., and Schweizer, H.P. (2006). mini-Tn*7* insertion in bacteria with single *att*Tn*7* sites: example *Pseudomonas aeruginosa*. *Nat. Protoc.* 1, 153-161. doi: 10.1038/nprot.2006.24

Hoang, T.T., Karkhoff-Schweizer, R.R., Kutchma, A.J., and Schweizer, H.P. (1998). A broad-host-range Flp-FRT recombination system for site-specific excision of chromosomally-located DNA sequences: application for isolation of unmarked *Pseudomonas aeruginosa* mutants. *Gene* 212, 77-86. doi: 10.1016/S0378-1119(98)00130-9

Li, K., Yang, G., Debru, A.B., Li, P., Zong, L., Li, P., et al. (2017). SuhB regulates the motile-sessile switch in *Pseudomonas aeruginosa* through the Gac/Rsm pathway and c-di-GMP signaling. *Front. Microbiol.* 8, 1045. doi: 10.3389/fmicb.2017.01045

Liberati, N.T., Urbach, J.M., Miyata, S., Lee, D.G., Drenkard, E., Wu, G., et al. (2006). An ordered, nonredundant library of *Pseudomonas aeruginosa* strain PA14 transposon insertion mutants. *Proc. Natl. Acad. Sci. U.S.A.* 103, 2833-2838. doi: 10.1073/pnas.0511100103

Stover, C.K., Pham, X.Q., Erwin, A.L., Mizoguchi, S.D., Warrener, P., Hickey, M.J., et al. (2000). Complete genome sequence of *Pseudomonas aeruginosa* PAO1, an opportunistic pathogen. *Nature* 406, 959-964. doi: 10.1038/35023079

West, S., Schweizer, H., Dall, C., Sample, A., and Runyen-Janecky, L. (1994). Construction of improved *Escherichia*-*Pseudomonas* shuttle vectors derived from pUC18/19 and sequence of the region required for their replication in *Pseudomonas aeruginosa*. *Gene* 148, 81-86. doi: 10.1016/0378-1119(94)90237-2
